# Molecular and cellular architecture of the larval sensory organ in the cnidarian *Nematostella vectensis*

**DOI:** 10.1242/dev.200833

**Published:** 2022-08-24

**Authors:** Eleanor Gilbert, Callum Teeling, Tatiana Lebedeva, Siffreya Pedersen, Nathan Chrismas, Grigory Genikhovich, Vengamanaidu Modepalli

**Affiliations:** 1 Marine Biological Association of the UK, The Laboratory, Citadel Hill, Plymouth PL1 2PB, United Kingdom; 2 School of Biological and Marine Sciences, University of Plymouth, Plymouth, PL4 8AA, UK; 3 Department of Neurosciences and Developmental Biology, Faculty of Life Sciences, University of Vienna, Vienna, 1030, Austria; 4 Doctoral School of Ecology and Evolution, University of Vienna, Vienna, 1030, Austria

**Keywords:** Apical organ, Neuron, Evolution, Cilia, Cnidaria, *Nematostella vectensis*

## Abstract

Cnidarians are the only non-bilaterian group to evolve ciliated larvae with an apical sensory organ, which is possibly homologous to the apical organs of bilaterian primary larvae. Here, we generated transcriptomes of the apical tissue in the sea anemone *Nematostella vectensis* and showed that it has a unique neuronal signature. By integrating previously published larval single-cell data with our apical transcriptomes, we discovered that the apical domain comprises a minimum of six distinct cell types. We show that the apical organ is compartmentalised into apical tuft cells (spot) and larval-specific neurons (ring). Finally, we identify *ISX-like* (*NVE14554*), a PRD class homeobox gene specifically expressed in apical tuft cells, as an FGF signalling-dependent transcription factor responsible for the formation of the apical tuft domain via repression of the neural ring fate in apical cells. With this study, we contribute a comparison of the molecular anatomy of apical organs, which must be carried out across phyla to determine whether this crucial larval structure evolved once or multiple times.

## Abstract (precis)

**Summary:** *ISX-like* homeobox gene is an FGF signalling-dependent transcription factor responsible for the formation of the apical tuft in the sea anemone *Nematostella vectensis*.

## INTRODUCTION

**Fig. 1.**
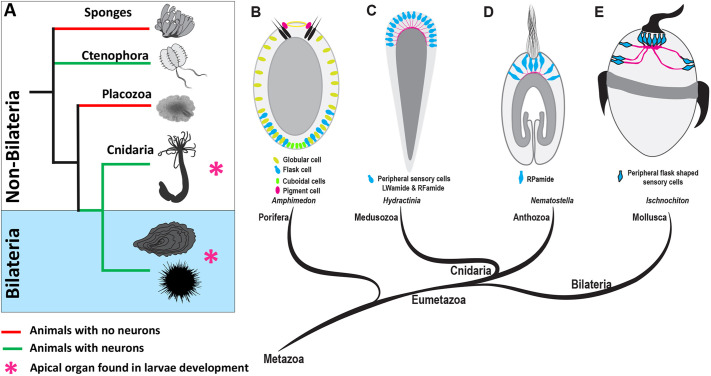
**Origin and evolution of the nervous system and the apical organ.** (A) A brief overview of evolutionary relationships in the animal kingdom. Porifera and Placozoa do not have defined neurons. Among non-bilaterians, Cnidaria and Ctenophora have well defined neurons. (B-E) Schematic drawings of surface-contacting flask-shaped cells in the ciliated larvae of different marine phyla. Sponge larvae (B) have ciliated photoreceptor cells capable of light sensing and other peripheral cell types such as flask and cuboidal cells. Among non-bilaterians (C,D), a true larval apical organ with integrated neurons is only found in cnidarians. Schematics were drawn based on the following primary data: (B) Nakanishi et al. (2015), Richards and Degnan (2012), Ueda et al. (2016); (C) Gajewski et al. (1996), Katsukura et al. (2004); (D) Zang and Nakanishi (2020); (E) Voronezhskaya et al. (2002), Nezlin and Voronezhskaya (2017). As ctenophore aboral sensory organs, which are sometimes also termed ‘apical organs’, are clearly not homologous to the larval apical organs of Cnidaria and Bilateria (Tamm, 2014; Edgar et al., 2022) we did not include them in this Figure.

During early development, the majority of marine benthic invertebrates progress through a planktonic life phase, consisting of a ciliated larva with an apical organ (Marinković et al., 2020). Several behavioural studies have demonstrated that ciliated larvae use the sensory organ at the apical pole to process environmental cues and modulate their swimming behaviour (Sinigaglia et al., 2015; Iwao et al., 2002; Schmich et al., 1998). The apical pole of the larvae is enriched with flask-shaped cells, usually with an apical tuft of non-motile cilia (Fig. 1) (Marinković et al., 2020; Conzelmann et al., 2011; Verasztó et al., 2017; Goldberg et al., 2011; Garner et al., 2016; Gruhl, 2009; Page, 2002; Chia and Koss, 1979). Besides the flask-shaped apical cells, the sensory (photosensitive and mechanosensory) and secretory/gland cells are also scattered around the apical pole and are likely associated with the sensory-ciliomotor nervous system. For instance, in bilaterian trochophore larvae, such as in larvae of the mollusc *Ischnochiton hakodadensis* (Fig. 1E) (Voronezhskaya et al., 2002; Nezlin and Voronezhskaya, 2017) and the annelid *Malacoceros fuliginosus*, the apical pole possesses several sensory cells that are positive for serotonin and the neuropeptide FMRFamide (Kumar et al., 2020). Similarly, the apical organ of the marine annelid *Platynereis dumerilii* is also equipped with photosensitive, mechanosensory and peptidergic cell types alongside an apical tuft (Marlow et al., 2014; Williams and Jékely, 2019; Randel et al., 2013; Verasztó et al., 2018).

Among non-bilaterians, a larval sensory organ with integrated neurons is only found in cnidarians (Fig. 1) (Kelava et al., 2015; Marlow et al., 2009; Layden et al., 2016). In anthozoans, like the sea anemone *Nematostella*, the apical pole displays several flask-shaped apical cells with a ciliated tuft and RPamide-positive sensory cells (Fig. 1D) (Zang and Nakanishi, 2020). In hydrozoans, the apical pole is highly enriched in cells expressing the neuropeptides LWamide and RFamide (Fig. 1C) (Piraino et al., 2011; Gajewski et al., 1996; Leitz and Lay, 1995; Katsukura et al., 2004). However, unlike anthozoans, the hydrozoans lack an apical organ-like sensory structure with a ciliated tuft (Pennati et al., 2013). Strikingly, animals devoid of neurons, such as the sponge *Amphimedon queenslandica*, also bear a set of sensory and secretory cells (globular/mucous cells) in the anterior region of the larvae (Nielsen, 2013; Brauchle et al., 2018), which are likely involved in modulating larval behaviour (Fig. 1B) (Woollacott, 1993; Richards and Degnan, 2012; Leys and Degnan, 2001; Maldonado et al., 2003). However, the advent of neuronal coordination of motile cilia is deemed to have been strategic in increasing the efficiency of sensory-to-motor transformation (Jékely, 2011).

The morphology of the apical organ in cnidarian larvae is comparable with that seen in bilaterian larvae (Sinigaglia et al., 2015; Marlow et al., 2014; Nielsen, 2015), indicating that the common ancestor of these two groups may have progressed through a free-swimming larval stage with a true larval apical organ and associated neurons (Nielsen, 2013, 2005). Earlier studies in *Nematostella* have identified a range of apical organ genes by blocking fibroblast growth factor (FGF) signalling during early development (Sinigaglia et al., 2015). Comparative gene expression studies between the cnidarian *Nematostella* and bilaterian ciliated larvae revealed a strong resemblance in the molecular topography around the apical pole (Marlow et al., 2014; Sinigaglia et al., 2013; Matus et al., 2006; Arendt et al., 2016), suggesting that the apical organ may be an evolutionarily conserved larval structure, although alternative hypotheses also suggest the convergent evolution of primary larvae (Liang et al., 2022 preprint).

**Fig. 2.**
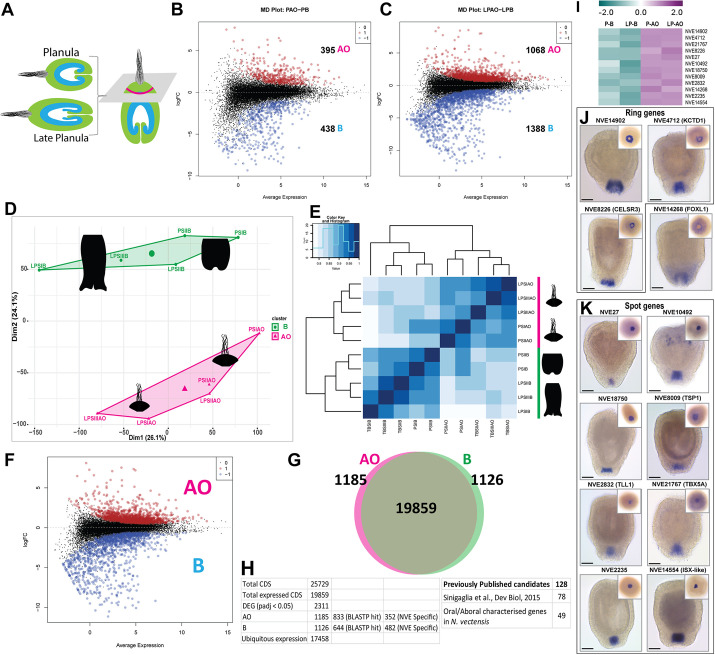
**Global transcriptomic analysis.** (A) Schematic representation of the apical region microdissected from the rest of the larval body tissue for transcriptomics. (B-C) Mean-difference (MD) plots represent the log[fold change (FC)] ratio of differential expression between apical and body tissues from (B) planula and (C) late planula stages. The upregulated and downregulated genes are highlighted as red and blue circles, respectively. Apical organ, AO; body, B; late planula apical organ, LPAO; late planula body, LPB; planula apical organ, PAO; planula body, PB. (D) PCA plot displaying a global overview of all datasets. (E) Correlation analysis identified two major clusters, as noted in the PCA plot. (F) MD plots represent the logFC ratio of differential expression between apical and body tissues. Datasets were pooled from planula and late planula development stages. (G) Venn diagram showing the DEGs from apical and body tissues. (H) A table detailing the number of DEGs in the current study, the number of genes with homologs and genes identified in the previous apical organ study by Sinigaglia et al. (2015), and the number of genes previously shown to be associated with the oral/aboral domains in *Nematostella* planula. For additional details, refer to Table S1. (I) A heatmap displaying the gene expression of selected marker genes enriched in apical organ cells. (J,K) ISH of apical organ-enriched genes. The insets show the apical view. Images are representative of approximately 40 *Nematostella* larvae per gene. Scale bars: 50 µm.

Cnidarians hold a key phylogenetic position for understanding nervous system evolution, and their larval sensory-ciliomotor nervous system provides a window to look into the primordial neurotransmission system. To advance understanding of this system, we aimed to map the apically enriched cell types and their gene expression profiles in the anthozoan cnidarian *Nematostella vectensis*. *Nematostella* is a well-established molecular model species that has been at the centre of fundamental discoveries in the development and evolution of the nervous system in non-bilaterian metazoans, making it a suitable model for the current study (Kelava et al., 2015; Marlow et al., 2009; Layden et al., 2016). Here, we reveal the gene expression profile of the larval apical domain (territory of the apical pole) including the sensory organ by performing transcriptomics on apical tissue that was separated from the rest of the larval body (Fig. 2A). Further, by integrating our tissue-specific (apical/body) transcriptome data with *Nematostella* larval single-cell RNA-sequencing (RNA-seq) data (Sebé-Pedrós et al., 2018), we identified the larval cell types enriched in the body and apical regions and their gene expression profiles. Finally, we identified *ISX-like* as an FGF-dependent transcription factor responsible for the definition of the apical tuft territory.

## RESULTS AND DISCUSSION

### Transcriptome profile of *Nematostella* apical sensory organ

We performed microdissections on *Nematostella* planula larvae and carefully separated the apical tissue from the rest of the larval body at the mid-planula (∼50-60 h post fertilisation) and late planula (∼75-85 h post fertilisation) developmental stages (Fig. 2A). We acquired transcriptome data from both the apical tissue and the rest of the body separately to perform differential gene expression (DGE) analysis. DGE analysis showed statistically significant variations among the apical and body tissue in both the mid-planula and late planula stages (Fig. 2B,C). The late planula stage (Fig. 2C) presented a relatively large number of differentially expressed genes (DEGs) in comparison with the mid-planula stage (Fig. 2B). Furthermore, to characterise global gene expression patterns among the apical and body tissues from the mid-planula and late planula stages, we compared the transcriptomic profiles of all datasets using principal component analysis (PCA) and correlation analysis (Fig. 2D,E). The plots displayed a strong correlation among the replicates and a significant difference between the apical and body datasets from both mid-planula and late planula stages. Notably, correlation analysis identified two major clusters and, as illustrated in the PCA plot, the apical datasets from both the planula and late planula formed a single cluster. Likewise, the body datasets fell under a single cluster irrespective of their developmental stage (Fig. 2F). Thus, for the downstream analysis, we pooled both apical datasets from the mid-planula and late planula developmental stages; likewise, we pooled the body datasets. A DGE analysis was carried out among the apical and body datasets to identify the significantly DEGs [adjusted *P*-value or *P*_adj_ (false discovery rate)<0.05]. We identified 2311 DEGs, of which 1185 were enriched in the apical domain and 1126 were enriched in the body (Fig. 2G,H; Table S1).

To validate our transcriptomic data by *in situ* hybridisation (ISH), we selected a set of newly identified apical pole-enriched genes from the DGE data (Fig. 2I). ISH showed that their expression is principally localised to the apical organ (Fig. 2J,K). Strikingly, two distinct expression profiles were observed: probes specific to *NVE14902* (*PoxA*), *NVE4712* and *NVE8226* (*Slc*) transcripts were localised around the apical cells (Fig. 2J), whereas probes specific to *NVE27*, *NVE10492*, *NVE8009*, *NVE2832*, *NVE2235* and *NVE14554* (*ISX-like*) transcripts (Fig. 2K) were localised specifically within the apical cells. This pattern was also identified with other *Nematostella* apical organ genes and termed as ‘spot’ and ‘ring’ (Sinigaglia et al., 2015). We then overlapped our RNA-seq dataset with the previously published list of 78 genes with confirmed aboral expression (Sinigaglia et al., 2015), and found that 71 out 78 were present among the 1185 aborally enriched transcripts we identified (Table S1).

### A distinct neuronal regulatory network associated with the larval apical region

**Fig. 3.**
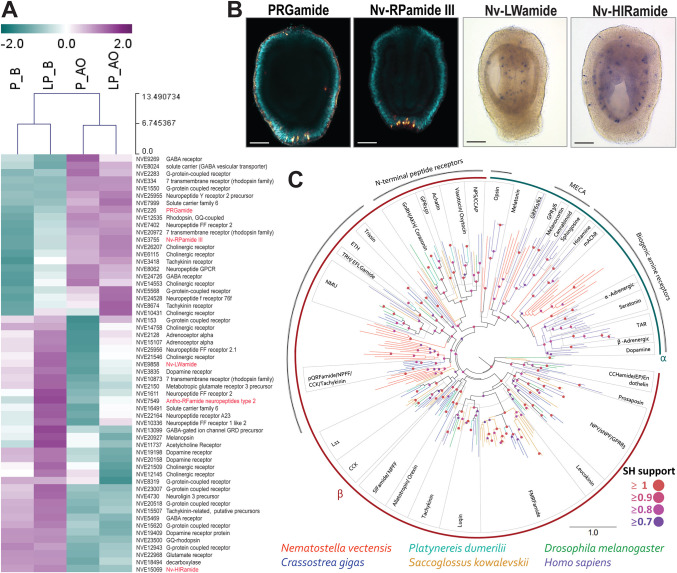
**Spatial distribution of larval nervous system-associated genes.** (A) Heatmap displaying the gene expression pattern of a set of genes related to diverse neuronal functions. Neuropeptide genes are highlighted in red. (B) Neuropeptides *Nv-RPamide III* and *PRGamide* were exclusively expressed in the apical tissue, whereas *Nv-LWamide* and *HIRamide* were detected predominantly in the body tissue. Scale bars: 50 µm. Images are representative of approximately 40 Nematostella larvae per gene. (C) Maximum likelihood analyses of the sequences from cluster analysis Fig. S2B. Phylogeny constructed with *C. gigas*, *P. dumerilii*, *S. kovalevski*, *D. melanogaster* and *H. sapiens .* SH support indicates fast approximate likelihood-based measures of branch supports. Adipokinetic hormone, AKH; crustacean cardioacceleratory peptide, CCAP; cholecystokinin, CCK; excitatory peptide, EP; ecdysis triggering hormone, ETH; gonadotropin releasing hormone, GnRH; muscarinic acetylcholine receptor, mAChR; neuromedin-U, NMU; neuropeptide F, NPF; neuropeptide S, NPS; neuropeptide Y, NPY; trace amine receptor, TAR; thyrotropin releasing hormone, TRH.

Neuropeptides in *Nematostella* may potentially be involved in modulating larval swimming behaviour like in bilaterians. For example, in annelid and echinoderm larvae, the ciliary beating frequency is modulated by neurotransmitters, and neuropeptides actively control the swimming speed by modulating the ciliary beating frequency (Conzelmann et al., 2011; Verasztó et al., 2017; Soliman, 1983, 1984). In ciliated larvae of the annelids *Platynereis* and *Capitella* as well as bryozoan *Cryptosula*, the neuropeptides are expressed in the peptidergic nerves that run along with the ciliary bands (Goldberg et al., 2011; Gruhl, 2009). In cnidarians, neuropeptides are expressed at the larval stage, and it was shown in hydrozoans that neurons expressing LWamide and RFamide are enriched in the aboral (anterior) pole of larvae (Gajewski et al., 1996; Leitz and Lay, 1995; Katsukura et al., 2004). We also noted a significant difference in the spatial distribution of neuropeptide transcripts along the oral-aboral axis in *Nematostella* (Fig. 3A). Along with the previously identified Nv-RPamide III neuropeptide (Zang and Nakanishi, 2020), we also detected PRGamide as a neuropeptide exclusively expressed in the apical tissue (Fig. 3A,B), whereas Nv-LWamide, Antho-RFamide neuropeptides type 2 and HIRamide were detected predominantly in the body tissue (Fig. 3A,B).

To understand the differences in neural gene expression between the apical domain and the body of the larva, we explored the tissue-specific transcriptomes using Gene Ontology (GO) analysis to identify genes related to neurotransmission. We performed a protein-protein Basic Local Alignment Search Tool (BLASTP) search in the UniProt database to identify the putative protein homologs. Additionally, we used gene functional annotation data from the published *Nematostella* single-cell transcriptome study (Sebé-Pedrós et al., 2018) (Table S1). Next, we analysed their GO terms using the David 6.7 and PANTHER 15.0 gene ontology tools. We identified several neuronal-associated genes that are differentially expressed between the apical and body regions, such as neuropeptides, G protein-coupled receptors (GPCRs), ligand-gated ion channels and neurotransmitter synthesis genes (Fig. 3A) (Fig. S1). This indicates that the neuromodulation in the apical region of the *Nematostella* larva is characterised by a specific set of genes and may differ from the rest of the body.

In bilaterian models, the neurotransmitters were shown to control the ciliary beating through two broad classes of receptors: ligand-gated ion channels and GPCRs (Goldberg et al., 2011; Soliman, 1983, 1984; Kuang et al., 2002; Lacalli et al., 1990). Notably, we observed a large number of GPCRs differentially expressed in the oral versus apical tissues (Fig. S1). GPCRs are the largest family of membrane receptors and mediate most of the cellular responses to hormones, neuropeptides and other neurotransmitters (Hewes and Taghert, 2001; Quiroga Artigas et al., 2020). To understand the relationship of apically enriched GPCRs with known GPCR families in Bilateria, we performed sequence-similarity-based clustering and maximum-likelihood phylogenetic analyses on apically enriched GPCRs, in parallel to BLASTP analysis. We used previously published datasets from *Crassostrea gigas*, *P. dumerilii*, *Saccoglossus kovalevski* and *Drosophila melanogaster* (Thiel et al., 2021), as well as *Homo sapiens* GPCRs (Quiroga Artigas et al., 2020). The *Nematostella* apically enriched GPCRs clustered with a range of GPCR superfamilies from bilaterians as shown in Fig. S2. Forty-six *Nematostella* apically enriched GPCRs clustered with a large group of rhodopsin and four clustered with secretin (Fig. S2). Within the rhodopsin superfamily, the GPCRs formed two major groups: rhodopsin α and β. Rhodopsin α GPCRs formed a monophyletic group which included 22 *Nematostella* GPCRs. The remaining 24 *Nematostella* GPCRs fell among the rhodopsin β clades (Fig. 3C). The rhodopsin α GPCRs can be further divided into the biogenic amine receptor group and the melanocortin, endothelial differentiation sphingolipid, cannabinoid and adenosine receptor (MECA) group that includes melanocortin and opsins. Among the rhodopsin β GPCRs, 23 *Nematostella* GPCRs formed three clades positioned among other neuropeptide GPCRs within the rhodopsin β superfamily. In summary, the current analysis allowed us to identify several orthologue groups of GPCRs expressed in the *Nematostella* apical domain and their distribution across different GPCR receptor families.

### Identifying the spatial distribution of *Nematostella* larval cell types by integrating tissue-specific transcriptomes with larval single-cell data

Fine morphological studies in different ciliated larvae (Chia and Koss, 1979; Pennati et al., 2013; Nakanishi et al., 2008; Martin and Chia, 1982) have revealed that, along with long ciliated apical cells, the apical region comprises other cell types such as neurons, gland cells and peripheral ciliated sensory cells. Identification of apical organ-associated cell types and their marker genes is strategic for understanding apical organ function. Single-cell RNA sequencing revealed transcriptome profiles of different cell types in ciliated larvae of *Nematostella* (Sebé-Pedrós et al., 2018). The larval cells were classified into 38 metacells, and each metacell represented a specific larval cell type with a unique transcriptome profile (Sebé-Pedrós et al., 2018). However, the spatial distribution of these cell types is yet to be addressed. To develop an atlas of apical organ-associated cell types and their transcriptomes, we integrated the tissue-specific transcriptome data with the *Nematostella* whole larval single-cell RNA-seq data (Sebé-Pedrós et al., 2018).

**Fig. 4.**
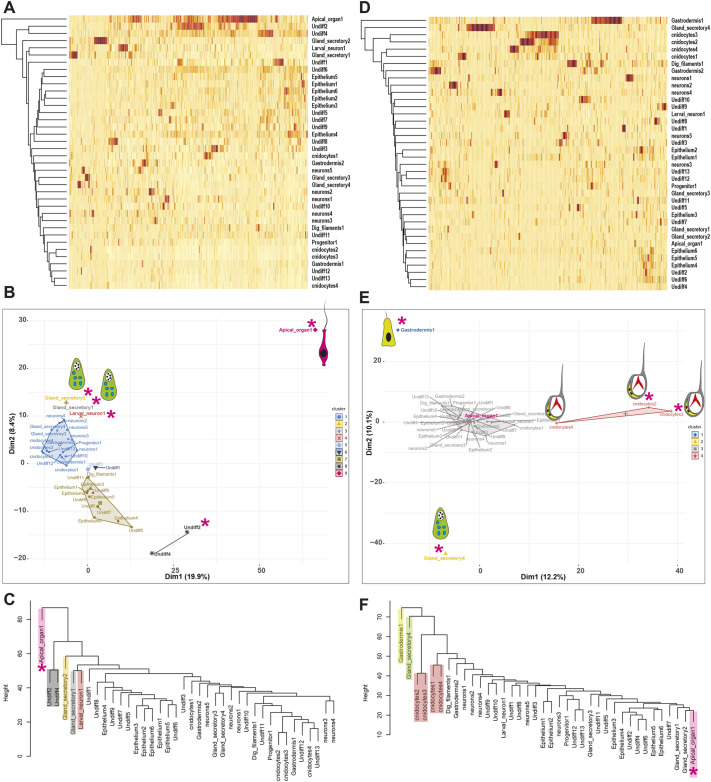
**Spatial distribution of *Nematostella* larval cell types.** (A,D) Expression of apically (A) and body- (D) enriched genes (rows) across 38 metacells sorted by cluster association. (B,E) Single-cell PCA plot for apically (B) and body- (E) enriched genes. Pink asterisks indicate apical organ cell type. (C,F) Hierarchical clustering for apically (C) and body- (F) enriched genes. The dendrogram produced similar results as single-cell PCA.

We analysed the expression profiles of apical pole-enriched genes (1185) in each larval metacell using cluster association (Fig. 4A), PCA (Fig. 4B) and hierarchical clustering (HC) (Fig. 4C) to define the metacells enriched in the apical domain. From the plots, we observed that the apical cell type (Apical_organ1) was clustered distinctly from other cell types and had a high expression of multiple apically enriched genes (Fig. 4A). Out of 38 metacells, the larva-specific neurons, gland/secretory-cell types 1 and 2, and undifferentiated cell types 2 and 4 also displayed specific expression of apically enriched genes (Fig. 4A), and stood out in the apical domain with minimum overlap with any other larval cell clusters (Fig. 4A-C). In parallel, we also analysed the expression profiles of the body-enriched genes (1126) in each larval metacell to identify the cells enriched in body tissue (Fig. 4D-F). In both PCA and HC, genes enriched in the body displayed a very distinctive trend from the apical tissue transcriptome (Fig. 4). We observed that the larval metacells including cnidocytes, gastrodermis and a specific gland/secretory-cell type 4 displayed high expression of body-enriched genes (Fig. 4D-F) and clustered distinctly from other metacells (Fig. 4E,F), suggesting that these cell types are likely spatially distributed in the body and devoid from the apical region.

### Flask-shaped gland/secretory cells enriched in the apical domain

**Fig. 5.**
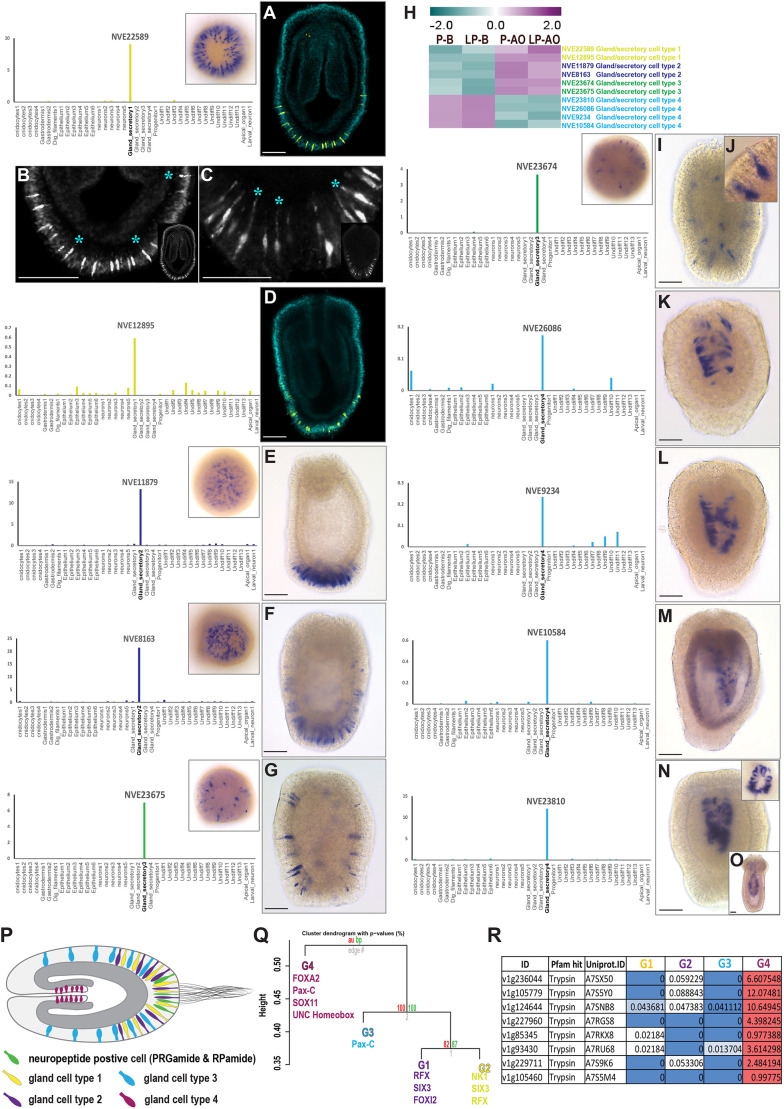
**Spatial distribution of larval gland/secretory-cell types visualised by whole-mount ISH.** (A,D-G,I-O) ISH (right) of larval gland/secretory-cell type marker genes. Each bar plot (left) displays the expression profile of selected marker genes in the different larval cell populations. The insets (top right) show the apical view. (A-D) ISH of larval gland/secretory-cell type 1. (B,C) Monopolar sensory cells with their projection from the cell body extended towards mesoglea (asterisks); the insets show the whole animal. (E,F) ISH of larval gland/secretory-cell type 2. (G,I,J) ISH of the larval gland/secretory-cell type 3; panel J shows the ciliated gland-cell type 3 at higher magnification. (H) Heatmap displaying the gene expression pattern of selected marker genes specific for each larval gland/secretory-cell types. (K-O) ISH of the larval gland/secretory-cell type 4; panel O demonstrates the spatial expression of gland-cell type 4 concentrated in the mesentery tissue. (P) Schematic representation of the spatial distribution of larval gland/secretory-cell types. (Q) Dendrogram displaying the correlation among gland cells and a list of a different combination of transcription factors expressed in each gland-cell type. (R) Gene expression of trypsin domain-containing proteins in gland cells from larval single-cell data. Images are representative of approximately 40 *Nematostella* larvae per gene. Scale bars: 50 µm.

Besides apical cells and larva-specific neuronal cell types, single-cell PCA revealed four gland/secretory-cell types with distinctive profiles among apical and body datasets (Fig. 4A-D). Out of four larval gland-cell types, cell types 1 and 2 were principally enriched in the apical region (Fig. 4A,C). In contrast, gland/secretory-cell type 4 was enriched exclusively in the body (Fig. 4E,F), whereas cell type 3 was expressed ubiquitously (Fig. 4). Recent studies have addressed the development of gland cells in *Nematostella*, mainly focusing on gland cells that develop and integrate into the pharynx and mesenteries of polyps (Steinmetz et al., 2017; Babonis et al., 2019), and ectodermal gland cells producing toxins (Sachkova et al., 2019; Columbus-Shenkar et al., 2018). However, not much focus has been given to other larval gland/secretory-cell types and their fate during development. To confirm the enrichment of gland/secretory-cell types in the apical domain, we selected specific marker genes expressed in each of these gland-cell types and visualised their expression pattern by ISH (Fig. 5). Gland cells type 1 and 2 were enriched in the apical region (Fig. 5A-F; Movie 1). Gland-cell type 3 was presented throughout the animal (Fig. 5G,I,J; Movie 2). The marker genes for cell types 1, 2 and 3 were expressed in the outer ectoderm of the planula (Fig. 5A-J). In contrast, marker genes (*NVE23810*, *NVE26086*, *NVE9234* and *NVE10584*) selected for gland-cell type 4 were expressed in the pharynx region and progressed into the pharyngeal/mesentery tissue of the primary polyp (Fig. 5K-O). As noted from ISH, gland-cell type 4 had a distinct expression from other peripheral gland cells and was principally restricted to pharyngeal/mesentery tissue (Fig. 5K-O). From the *Nematostella* larval single-cell data, we noted that the trypsin domain-containing proteins were restricted to gland-cell type 4 (Fig. 5R), suggesting that gland cell 4 is the larval cell type that develops into polyp gland/secretory-cell types in the mesenteries, where the digestive enzymes like trypsin are synthesised and secreted into the gastrovascular cavity for digestive function (Babonis et al., 2019).

To gain further insights into molecular features of the larval gland-cell types, we looked into the *Nematostella* larval single-cell data (Sebé-Pedrós et al., 2018). We noted that each of these gland cells are regulated through a set of transcription factors and some are unique to specific gland-cell types (Fig. 5P,Q). For instance, gland-cell type 1 expresses *retinal homeobox* (*Rx*) (*v1g184843*) and *FoxQ2d* (*v1g96685*), and gland-cell type 2 expresses *Emx1* (*v1g8907*). However, some of the transcription factors are expressed in several gland-cell types. For example, *PaxC* (*v1g168908*) is expressed both in gland-cell types 3 and 4. Similarly, *RFX4* (*v1g122918*) is expressed in both gland-cell types 1 and 2 (Fig. 5Q). This suggests that a different combination of transcription factors modulates the trajectory of each of these gland cells.

### *Nematostella* apical organ is composed of apical tuft cells crowned with larval neurons

Based on the *Nematostella* single-cell transcriptome study (Sebé-Pedrós et al., 2018), two metacells were classified as larval specific with low similarity to any adult cell cluster. Based on the previously identified marker genes *Fgf1a* (Sinigaglia et al., 2015) and *Nk3*, one of these metacells was recognised as an apical cell type. The second metacell showed a peculiar expression of genes with putative neuronal functions such as *cyclic nucleotide-gated*, *TrpA*, *polycystic kidney disease* and *shaker* ion channels; therefore, it was classified as an uncharacterised larval-specific neuronal cell type (Sebé-Pedrós et al., 2018).

**Fig. 6.**
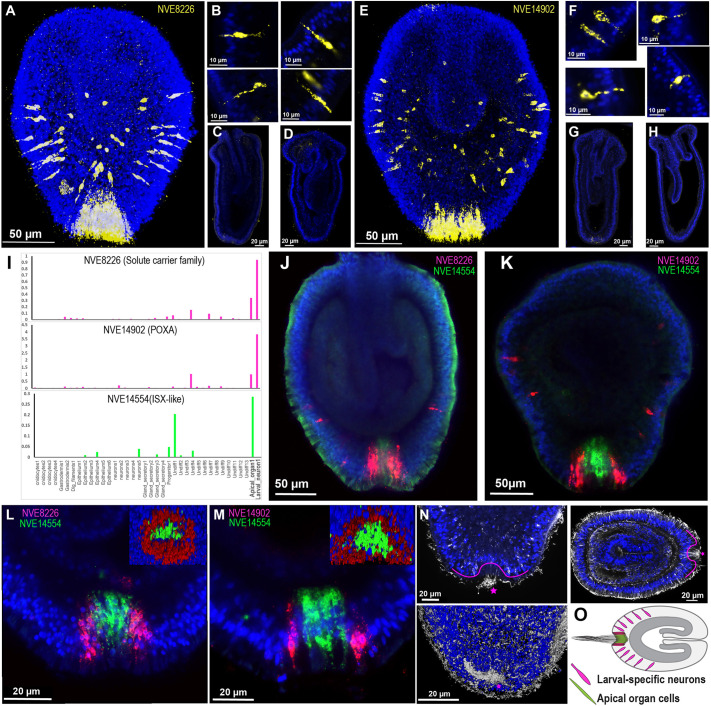
**Expression analysis of apical spot and neuronal ring markers.** (A-D) FISH analysis of *NVE8226* (yellow). (E-H) FISH analysis of *NVE14902* (yellow). A,B,E,F show the planula, C,G show metamorphosis and D,H show the early primary polyp. (I) Bar plot displaying the expression profiles of selected marker genes. (J-M) Double FISH demonstrates mutual localisation of marker genes for the spot (*ISX-like*; green) and the ring (*NVE8226* and *NVE14902*; red). DAPI-stained nuclei are shown in blue. The insets (L,M) on the top right display the three-dimensional image from the apical view. (N) Immunostaining with the anti-acetylated tubulin antibody (white) counterstained with DAPI (blue) for nuclei; the pink line demonstrates the apical tuft cells concentrated in the apical pit. Pink stars indicate the apical tuft. J-N show the planula. Images are representative of approximately 40 *Nematostella* larvae per gene. (O) Summary diagram showing the spatial distribution of the apical organ/tuft and larval-specific neuronal cell types.

From single-cell PCA and HC analyses, we observed that along with the apical cell type, the larva-specific neurons stood out in the apical domain (Fig. 4A-C). In our initial ISH analysis of apically enriched genes, we observed two distinct expression profiles: a set of genes expressed around the apical cells, and others expressed specifically in apical cells (Fig. 2J). The distinct expression patterns of apical organ genes suggest that apical sensory structure is composed primarily of two distinct cell types. Based on the *Nematostella* single-cell transcriptome (Sebé-Pedrós et al., 2018), the marker genes identified as spot specific were principally restricted to the apical cell type (Fig. 6I). However, the marker genes expressed as a ring were enriched in larval neurons (Fig. 6I). To explore this further, we carried out double fluorescence *in situ* hybridisation (FISH) on these marker genes (Fig. 6). The *NVE8226* and *NVE14902* markers, besides being expressed as a ring around the apical cells, showed expression across the whole apical region (Fig. 6A,E; Movies 3 and 4). These cells have a peculiar sensory neuronal morphology (Marlow et al., 2009) and are localised in the ectoderm with the tip pointing towards the periphery (Fig. 6B,F). Expression of these genes gradually diminishes as the larvae progress through metamorphosis (Fig. 6C,D,G,H), likely reflecting their larval-specific function.

Based on the double FISH, the apical organ gene *ISX-like* was found to be restricted to the apical pit and crowned with larva-specific neurons (detected by *NVE8226* and *NVE14902* markers) (Fig. 6J-M; Movies 5 and 6). The cells carrying the apical tuft cilia, which were visualised by immunostaining with an anti-acetylated tubulin antibody, were concentrated in the apical pit, where the spot genes like *ISX-like* are expressed (Fig. 6N). Our analysis allowed us to localise the previously uncharacterised larval-specific neuronal cell type to the apical organ ring. This localisation suggests that the apical cells with their ciliated tufts act as a sensory structure, and the larva-specific neurons receive information by crowning around them and probably signalling downstream to the rest of the body (Fig. 6O). The apical organ cells lack any recognisable neuronal effector genes such as GPCRs, synaptic scaffold proteins, neurotransmitter-related enzymes and neuropeptides, suggesting a non-neuronal identity (Sebé-Pedrós et al., 2018). However, the larval-specific neuronal cell type is enriched with neuronal functional genes. To conclude, the apical tuft cells and larval-specific neurons are the principal cell types in the apical sensory organ (Fig. 6O).

### *ISX-like* is responsible for the formation of the apical tuft cell fate

*ISX-like* encodes a paired-class homeodomain transcription factor with a typical spot expression in the apical organ (Figs 3 and 4). It was one of the prominent validated DGE targets in our screen and was previously described as an aborally expressed gene negatively regulated by β-catenin signalling (Lebedeva et al., 2021). Earlier studies (Mazza et al., 2010; Brauchle et al., 2018) classified *ISX-like* as a PRD class homeobox gene. Reciprocal BLAST search and phylogenetic analysis (Fig. S3) showed that *ISX-like* is related to the bilaterian PRD class *intestine-specific homeobox* (*ISX*). *Nematostella ISX-like* starts to be expressed in a broad aboral domain around 14 h post fertilisation (Fig. S4A), i.e. 2 h after the onset of the expression of the key aboral regulator *Six3/6* (Sinigaglia et al., 2015; Lebedeva et al., 2021), and later becomes restricted to a spot domain in the apical tuft cells (Fig. 6J-M). As our FISH analyses showed that the *ISX-like*-expressing apical tuft cells and the ring of circumapical neurons formed two perfectly complementary domains, we asked whether *ISX-like* was involved in the regulation of the development of the spot and the ring.

**Fig. 7.**
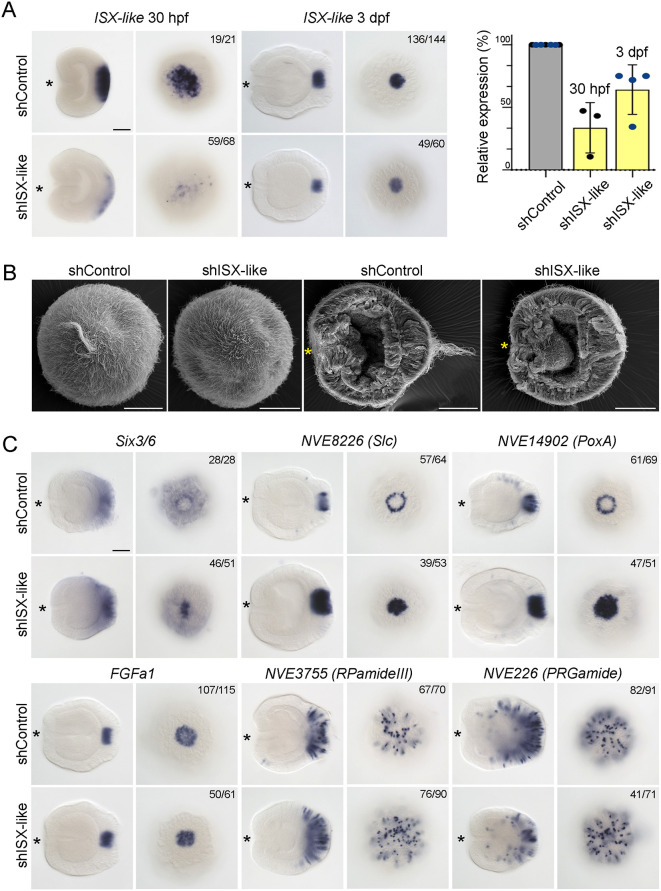
***ISX-like* RNAi efficiency and loss-of-function analysis of *ISX-like*.** (A) At 30 hpf, *ISX-like* RNAi efficiently reduces the amount of *ISX-like* mRNA as shown by ISH (left) and qPCR (right). By 3 dpf, *ISX-like* expression largely recovers. Data show the mean±s.d. (B) *ISX-like* RNAi results in loss of the apical tuft. SEM of 72 hpf embryos viewed aborally (two images on the left) or laterally (two images on the right; the embryos were cracked open to visualise the internal structures). (C) Effect of *ISX-like* RNAi on marker gene expression in the 3 dpf planula. Ring genes *NVE8226* and *NVE14902* became spot genes. *PRGamide* and *RPamide III* expression was not affected. *Six3/6* expression extended into the apical organ. *FGFa1* expression was not affected. Lateral and aboral views are shown. For lateral views, asterisks denote the oral end. In A and C, the numbers in the top right corner show the fraction of the embryo demonstrating this phenotype. B,C show the planula. Scale bars: 50 µm.

RNAi-mediated, as well as antisense morpholino-mediated knockdown of *ISX-like* resulted in the complete loss of the apical tuft (Fig. 7A,B; Fig. S5). Analysis of the morphants showed that the fraction of larvae successfully undergoing metamorphosis was moderately reduced in comparison with controls (68% upon *ISX-like* knockdown versus 82% in control; unpaired, two-tailed *t*-test, *t*=3.71391, *P*=0.020584) (Fig. S5). ISH analyses showed that *ISX-like* knockdown did not simply prevent the formation of the apical tuft cilia, but rather led to a loss of the apical tuft cell identity, i.e. the expression of the larva-specific ring neuron markers *NVE8226* and *NVE14902*, as well as the expression of the upstream aboral regulator *Six3/6*, expanded into the spot domain (Fig. 7C; Fig. S5). In contrast, the spot expression of the aborally enriched neuropeptide genes *PRGamide* (*NVE226*) and *RPamide III* (*NVE3775*) was not affected at all (Fig. 7C; Fig. S5). Thus, we conclude that the formation of the apical tuft cells takes place due to *ISX-like*-dependent repression of the neural ring fate in these cells.

**Fig. 8.**
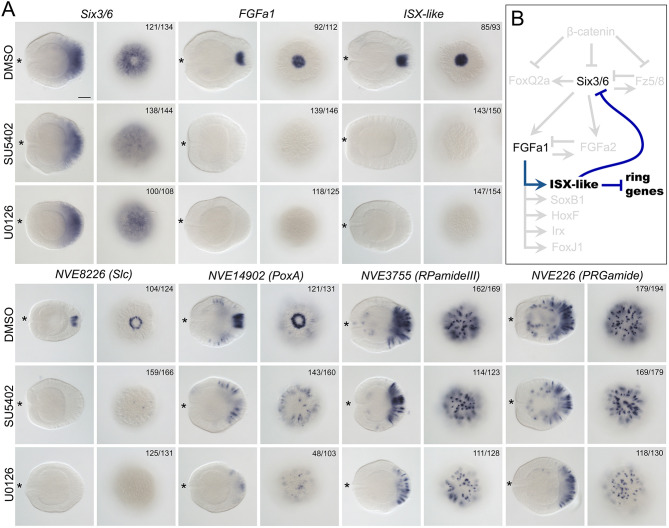
***ISX-like* expression is controlled by FGF signalling.** (A) The expression of the spot and ring genes is abolished upon FGF receptor or MEK inhibition, but not the expression of the aborally enriched *PRGamide* and *RPamide III*. *Six3/6* expression extends into the apical organ domain. Lateral and aboral views of 3 dpf planulae are shown. For lateral views, asterisks denote the oral end. The numbers in the top right corner show the fraction of the embryos displaying the phenotype shown on the image out of the total number of embryos treated and stained as indicated on the figure. Scale bar: 50 µm. (B) Genetic interactions regulating the apical domain patterning based on findings by Sinigaglia et al. (2013) and Leclère et al. (2016) (grey) and this paper (black and blue).

As the formation of the apical tuft was previously shown to be FGFa1-dependent (Rentzsch et al., 2008), we then tested whether *ISX-like* was upstream or downstream of FGF signalling. Although *FGFa1* expression appeared to be only very weakly affected by *ISX-like* knockdown (Fig. 7C; Fig. S5), the expression of *ISX-like* was abolished upon incubation of the embryos in the FGF receptor inhibitor SU5402 and MEK inhibitor U0126, suggesting that *ISX-like* expression was positively controlled by FGF signalling (Fig. 8A). Surprisingly, the expression of the neural ring markers *NVE8226* and *NVE14902* reacted differently to SU5402 and U0126 treatment. Although *NVE8226* expression was abolished upon treatment with both inhibitors, the ring expression of *NVE14902* was abolished upon treatment with the FGF receptor inhibitor SU5402 and in approximately 61% (*n*=103) of the embryos treated with the MEK inhibitor U0126 (Fig. 8A; Fig. S6). However, *NVE14902* showed spot expression in the remaining 39% of the embryos treated with U0126 (Fig. S6), which suggests a more complex, yet unclear type of regulation, possibly by non-FGF-mediated MAPK signalling. Expression of the aborally expressed neuropeptide genes *PRGamide* and *RPamide III* was not abolished by treatment with either of the inhibitors, although the expression appeared weaker in U0126-treated embryos. Taken together, we conclude that *ISX-like* is an FGF signalling-dependent transcription factor responsible for the formation of the apical tuft domain acting via repression of the neural ring fate in apical cells.

### Conclusions and outlook

Despite the obvious similarity to bilaterian apical organs, the *Nematostella* apical organ remains a mysterious structure. Combined with the previously published single-cell data (Sebé-Pedrós et al., 2018), our study provides an entry point to explore its function. Contrary to a previous assumption (Marlow et al., 2014), the *Nematostella* planula single-cell transcriptome suggests that apical tuft cells lack recognisable neuronal effector transcripts, suggesting a non-neuronal identity. Conversely, the larval-specific neuronal cell cluster is enriched with neuronal transcription factors such as *foxQ2b* and *soxB(2a)*, and ion channels implicated in putative neuronal functions (Sebé-Pedrós et al., 2018). In this study, we revealed that apical tuft cells are crowned with larval-specific neurons, suggesting that these cell types together form the apical organ of the *Nematostella* planula. For the apical organ (i.e. the apical tuft cells plus the neural ring cells) to be considered a true sensory organ, future research will have to demonstrate the transmission of information from the apical tuft cells to the larval nervous system. The formation of the apical organ is FGF-dependent: loss of the apically expressed *FGFa1* or its putative receptor *FGFRa*, as well as pharmacological inhibition of FGF signalling in *Nematostella* blocks development of the apical tuft (Rentzsch et al., 2008) and the neural ring (Fig. 8). Moreover, the metamorphosis of the *Nematostella* planula into polyps is also suppressed by *FGFa1* and *FGFRa* knockdown, as well as by pharmacological inhibition of FGF signalling (Rentzsch et al., 2008). Although it is tempting to think that the loss of the apical tuft may be the cause of the failed metamorphosis, our data do not support this hypothesis: knockdown of the FGF-dependent transcription factor ISX-like results in a complete loss of the apical tuft domain in the planula, but the effect on metamorphosis is very mild (Figs. 4 and 7). In the future, it will be important to functionally test the potential role of the neural ring cells of the apical organ, as well as of the peptidergic and gland/secretory cells enriched in the apical domain, in driving metamorphosis.

Another more profound question is the evolutionary origin of apical organs and whether the apical organs of ciliated larvae across different phyla are homologous or evolved convergently. The widespread occurrence of ciliated larvae with apical organs has prompted radically different views on their evolutionary significance. Some authors consider ciliated larvae to represent the ancestral metazoan morphology (Jagersten, 1972; Nielsen, 2012; Peterson et al., 1997; Davidson et al., 1995), whereas others strongly suggest that larval stage was intercalated independently multiple times in phylogenetically distinct lineages (Liang et al., 2022 preprint; Raff, 2008; Sly et al., 2003; Wolpert, 1999). Naturally, the homology of apical organs becomes questionable if primary larvae evolved convergently in different phyla. However, although the ‘adult first versus larva first’ question remains debated, a highly conserved set of genes patterning the apical/anterior ectoderm in directly and indirectly developing Bilateria and in the apical/aboral ectoderm in Cnidaria shows that these regions are very likely homologous (Marlow et al., 2014; Sinigaglia et al., 2013; Lebedeva et al., 2021; Range et al., 2013; Kitzmann et al., 2017; Santagata et al., 2012). This, in turn, makes it important to consider the possible ‘deep homology’ of the apical organs, even if primary larvae in different phyla evolved convergently, and to analyse the relationship between the cell types building the apical organs across phyla by using transcriptomic methods. Our study using an anthozoan cnidarian model makes a step in this direction and provides a valuable set of data from a member of the bilaterian sister group.

## MATERIALS AND METHODS

### Nematostella culture

*Nematostella* polyps were grown in 16‰ artificial seawater at 18°C in the dark and fed with freshly hatched *Artemia* nauplii. The induction of spawning was performed as previously described (Genikhovich and Technau, 2009a). After fertilisation, the gelatinous substance around the eggs was removed using 4% L-cysteine (Sigma-Aldrich) (Genikhovich and Technau, 2009a).

### Microdissection of *Nematostella* apical organs

We performed microdissection on *Nematostella* larvae to separate the apical organ from the rest of the larval body. Two developmental stages including planula and late planula were used. Apical tissue containing the apical organ (see Fig. 2A) was isolated using 34-gauge needles under a stereomicroscope with 10× magnification. Motile larvae were placed into a fresh plastic Petri dish filled with *Nematostella* medium. The larvae tend to adhere briefly to the bottom of a fresh plastic Petri dish, allowing enough time to separate the apical tissue by cutting. Each sample was pooled from a minimum of 50 individual larvae and also included samples from a minimum of three different batches. The samples were carefully collected using glass Pasteur pipettes, excess medium was removed, and the samples were snap-frozen in liquid nitrogen and stored at −80°C until further processing.

### RNA sequencing and differential gene expression

For RNA isolation, the tissue samples collected from multiple batches were combined to acquire an adequate amount of RNA for sequencing. Total RNA was isolated using the TRI Reagent (Sigma-Aldrich) according to the manufacturer's protocol. RNA quality was assessed using an Agilent RNA 6000 Nano Kit on an Agilent 2100 Bioanalyzer, and samples with RNA integrity number ≥8.0 were used for sequencing. The SENSE mRNA-Seq Library Prep Kit (Lexogen) was used for library preparation. Before sequencing, the libraries were pre-assessed by Agilent High Sensitivity DNA Kit (Agilent) and quantified using Qubit 1× dsDNA HS Assay Kit (Invitrogen). The sequencing was outsourced (GENEWIZ Illumina NovaSeq/HiSeq 2×150 bp sequencing), generating 20 million paired end reads per replicate. Raw data were deposited at the Gene Expression Omnibus with the accession ID GSE159166. After de-multiplexing and filtering high-quality sequencing reads, the adapter contamination was removed by using Trimmomatic v0.36 (Bolger et al., 2014). The quality of the reads was verified using FastQC (http://www.bioinformatics.babraham.ac.uk/projects/fastqc). Processed reads from each sample were mapped to the *Nematostella* genome (indexed bowtie2; Langmead and Salzberg, 2012) by using STAR (Spliced Transcripts Alignment to a Reference) (Steinmetz et al., 2017). The number of reads mapping to each *Nematostella* gene model (https://figshare.com/articles/Nematostella_vectensis_transcriptome_and_gene_models_v2_0/807696) was extracted using HTSeq-count v0.6 (Babonis et al., 2019). Differential expression analyses were performed using limma (Galaxy version 2.11.40.6) (Love et al., 2014) and DESeq2 (Galaxy version 2.11.40.6) (Love et al., 2014). PCA, HC and heat maps were generated using the R package in R-studio (version 1.2.5019). For functional annotation, we used BLASTP (Altschul et al., 1997) with the default curated gathering threshold to predict the protein homologs against the UniProt database. Additionally, we used gene functional annotation data from a published *Nematostella* single-cell transcriptome study (Sebé-Pedrós et al., 2018). GO term enrichment was performed using gene annotation tools including PANTHER Classification System (Mi et al., 2012) and DAVID (Huang da et al., 2009).

### ISH and double FISH

ISH was performed according to published protocols (Wolenski et al., 2013; Genikhovich and Technau, 2009b). In brief, fixed animals were transferred into sieves and rehydrated in 1 ml 60% methanol/40% PBST [1× PBS with 0.05% (vol/vol) Tween-20] and then washed in 30% methanol/70% PBST. Samples were digested in proteinase K (80 µg/ml, Ambion) for 5 min, then blocked in glycine (4 mg/ml). Larvae were then transferred into 4% formaldehyde at room temperature (RT) for 1 h. Hybridisation was carried out with DIG-labelled probes for 48 h at 60°C. After incubation, samples were washed through serial dilutions of 25%, 50%, 75% and 100% with 2× SSCT buffer (20× concentrate, contains 0.3 M sodium citrate in 3 M NaCl, pH 7.0) at hybridisation temperature. The colour development was carried out in a 1:50 dilution of nitro blue tetrazolium/5-bromo-4-chloro-3-indolyphosphate (NBT/BCIP, Sigma-Aldrich) at RT. Stained animals were visualised with a Leica DM1000 microscope equipped with a MC190 HD microscope camera (Leica, Germany). For each gene, at least 30 specimens were tested. For double FISH after the SSCT washes, samples were blocked in 0.5% blocking reagent (FP1020, PerkinElmer) at RT for 1 h. Samples were then incubated overnight with anti-digoxigenin (1:100; Roche). After TNT (0.1 M Tris-HCl pH 7.5,0.15 M NaCl, 0.5% Triton X-100) washes, samples were incubated in Cy3 (NEL744001KT, TSA Plus Kit, PerkinElmer). To stop the peroxidase activity, the samples were washed in 0.1 M glycine, pH 2.0, and then incubated overnight with anti-fluorescein (1:250; Roche). Samples were then washed in TNT and incubated with DAPI (1:1000). Samples were imaged on Leica TCS SP8 DLS confocal microscope.

### Whole-mount immunofluorescence and scanning electron microscopy

After 1 h of fixation in 3.7% formaldehyde in *Nematostella* medium (16‰ artificial seawater), the samples were washed five times with PBST for 10 min. The samples were blocked in 5% bovine serum albumin (BSA) in PBST for 1 h at RT. Primary antibody (1:500, mouse anti-α-tubulin, T9026, Sigma-Aldrich) incubation was performed in a blocking solution (1% BSA in PBST) for 24-36 h at 4°C. The samples were washed with PBST five times for 5 min, after which samples were incubated with secondary antibodies (1:250, goat anti-mouse IgG Alexa Fluor 594, A-11032, Thermo Fisher Scientific) diluted in blocking solution overnight at 4°C. Then, the samples were washed with PBST five times for 10 min. Imaging was performed on Leica TCS SP8 DLS and Leica DMi8 confocal microscopes. Sample preparation for scanning electron microscopy (SEM) was performed as in Kraus et al. (2016); SEM imaging was performed using the JEOL IT 300 scanning electron microscope.

### GPCR clustering and phylogeny

We used previously published GPCR datasets of *Crassostrea virginica*, *P. dumerilii*, *S. kovalevski* and *D. melanogaster* (Thiel et al., 2021), as well as *H. sapiens* GPCRs (Quiroga Artigas et al., 2020). Initially, to identify the *Nematostella* GPCR candidates, grouping with bilaterians was done by clustering analysis using CLANS (Frickey and Lupas, 2004) with the BLOSUM62 scoring matrix and a *P*-value cut-off of 10^-25^. Sequences from the main cluster were used in phylogenetic analysis. Sequences were aligned with MUSCLE (Edgar, 2004) using default settings and trimmed with TrimAl using the automated mode (Capella-Gutierrez et al., 2009). Maximum likelihood phylogenetic analyses were completed using PhyML 3.0 online (www.atgc-montpellier.fr/phyml/) (Guindon et al., 2010). The model LG +G +F was automatically selected by the Smart Model Selection with (SH-aLRT).

### Gene knockdown and inhibitor treatment

Preparation of the short hairpin RNA against the target sequence 5′-GCGCTAGTCAACATACTGA-3′ and RNAi of *ISX-like* was performed by electroporation as described by Karabulut et al. (2019). Microinjection of the translation-blocking morpholino oligonucleotide 5′-AATTCTCTGATTTTTCCATCGTGG-3′ was used as the second independent approach to knock down *ISX-like*. As controls, shRNA against mOrange (Lebedeva et al., 2021) and a previously described control morpholino (Kraus et al., 2016) were used. RNAi efficiency was tested by quantitative PCR (qPCR) using the primers nve14554F_q (5′-ATGGAGCGAGTGTTTCTGCT-3′) and nve14554R_q (5′-CTTGCGCCATTTAGCTCTTC-3′), and the activity of the morpholino was checked by co-injecting it with the wild-type or the 5-mismatch mRNA containing the morpholino recognition sequence fused in-frame to the mCherry-coding sequence (Fig. S4B). Capped mRNA was synthesised using mMessage mMachine kit (Life Technologies) and purified with the Monarch RNA clean-up kit (New England BioLabs). For the inhibitor treatment, stock solutions of U0126 (Sigma-Aldrich) and SU5402 (Sigma-Aldrich) in DMSO were diluted to a final concentration of 15 µM and 20 µM in 16‰ artificial seawater, respectively. For DMSO controls, we used the same volume as for U0126, which was diluted 3:1000 from a less concentrated stock solution, which corresponds to a final DMSO concentration of 42.3 mM. The treatment lasted from 30 h post fertilisation (hpf) until 3 days post fertilisation (dpf).

## Supplementary Material

Supplementary information

Reviewer comments
