# Supplementary material for: Molecular and cellular architecture of the larval sensory organ in the cnidarian Nematostella vectensis
Source: Development. 2022 Aug 24;149(16):dev200833. doi: 10.1242/dev.200833 (PMC9481973; doi:10.1242/dev.200833)
Supplement: Supplementary information [file develop-149-200833-s1.pdf]

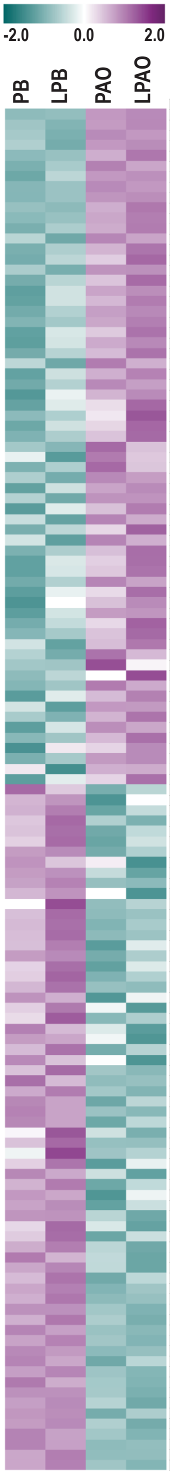


| NVE_GeneId | Gene ID | Symbol | Gene Name | Protein Class | GO |
|---|---|---|---|---|---|
| NVE8371 | MOUSE\|MGI\|MGI\|108078\|\|P97299 | SFRP2 | Secreted frizzled-related protein 2 | transmembrane signal receptor | transmembrane signal receptor (PC00197) |
| NVE10382 | FELCA\|Ensembl\|ENSFCAG00000025878\|\|M3X1M2 | HMCN1 | Uncharacterized protein | - | neuron projection (GO:0043005) |
| NVE19736 | BOVIN\|Ensembl\|ENSBTAG00000020957\|\|F1MGM1 | FZD5 | Frizzled class receptor 5 | transmembrane signal receptor | transmembrane signal receptor (PC00197) |
| NVE10381 | ANOCA\|Ensembl\|ENSACAG00000008287\|\|H9GE51 | NCAM1 | Uncharacterized protein | - | neuron projection (GO:0043005) |
| NVE6997 | BOVIN\|Gene\|HMCN1\|\|E1BE11 | HMCN1 | Uncharacterized protein | tyrosine protein kinase receptor | transmembrane signal receptor (PC00197) |
| NVE8133 | MOUSE\|MGI\|MGI\|2441950\|\|Q80TS3 | AGRL3 | Adhesion G protein-coupled receptor L3 | G-protein coupled receptor | transmembrane signal receptor (PC00197) |
| NVE15843 | MOUSE\|MGI\|MGI\|1860130\|\|Q924H0 | NPFF2 | Neuropeptide FF receptor 2 | G-protein coupled receptor | transmembrane signal receptor (PC00197) |
| NVE23855 | HUMAN\|HGNC\|13800\|\|Q9GZZ6 | ACH10 | Neuronal acetylcholine receptor subunit alpha-10 | ligand-gated ion channel | neuron projection (GO:0043005) |
| NVE2185 | CAEBR\|Gene\|CBG01491\|\|A8WQK3 | ACH1 | Acetylcholine receptor subunit alpha-type acr-16 | ligand-gated ion channel | neuron projection (GO:0043005) |
| NVE26112 | ANOCA\|Ensembl\|ENSACAG00000012709\|\|H9GJH9 | EPHA5 | Uncharacterized protein | - | neuron projection (GO:0043005) |
| NVE4515 | HUMAN\|HGNC\|14006\|\|Q9H1D0 | TRPV6 | Transient receptor potential cation channel subfamily V member 6 | ion channel | neuron projection (GO:0043005) |
| NVE22264 | DANRE\|ZFIN\|ZDB-GENE-081022-145\|\|B3DHM6 | ADRB1 | Adrenergic, beta-1-, receptor | G-protein coupled receptor | transmembrane signal receptor (PC00197) |
| NVE4175 | BOVIN\|Gene\|HMCN1\|\|E1BE11 | HMCN1 | Uncharacterized protein | tyrosine protein kinase receptor | transmembrane signal receptor (PC00197) |
| NVE3518 | BOVIN\|Gene\|SCNN1A\|\|P55270 | SCNNA | Amiloride-sensitive sodium channel subunit alpha | ion channel | neuron projection (GO:0043005) |
| NVE5022 | HUMAN\|HGNC\|11048\|\|P23975 | NET1 | Sodium-dependent noradrenaline transporter | primary active transporter | neuron projection (GO:0043005) |
| NVE5065 | CAEEL\|WormBase\|WBGene00001519\|\|Q9U7D5 | ACM3 | Muscarinic acetylcholine receptor gar-3 | G-protein coupled receptor | neuron projection (GO:0043005) |
| NVE7261 | DROME\|FlyBase\|FBgn0053517\|\|Q8IS44 | DRD2L | Dopamine D2-like receptor | G-protein coupled receptor | transmembrane signal receptor (PC00197) |
| NVE15552 | ANOCA\|Ensembl\|ENSACAG00000013826\|\|G1KPD2 | SYT1 | Uncharacterized protein | membrane trafficking regulatory protein | neuron projection (GO:0043005) |
| NVE1646 | BOVIN\|Gene\|CHRNA7\|\|P54131 | ACHA7 | Neuronal acetylcholine receptor subunit alpha-7 | ligand-gated ion channel | neuron projection (GO:0043005) |
| NVE17372 | HUMAN\|HGNC\|5302\|\|P34969 | 5HT7R | 5-hydroxytryptamine receptor 7 | G-protein coupled receptor | neuron projection (GO:0043005) |
| NVE13969 | CAEEL\|WormBase\|WBGene00006765\|\|P48181 | ACH2 | Acetylcholine receptor subunit beta-type unc-29 | ligand-gated ion channel | neuron projection (GO:0043005) |
| NVE4045 | HUMAN\|HGNC\|13800\|\|Q9GZZ6 | ACH10 | Neuronal acetylcholine receptor subunit alpha-10 | ligand-gated ion channel | neuron projection (GO:0043005) |
| NVE21238 | BOVIN\|Gene\|HMCN1\|\|E1BE11 | HMCN1 | Uncharacterized protein | tyrosine protein kinase receptor | transmembrane signal receptor (PC00197) |
| NVE10029 | BOVIN\|Gene\|BSG\|\|Q865R3 | BASI | Basigin (Fragment) | scaffold/adaptor protein | neuron projection (GO:0043005) |
| NVE21441 | ANOCA\|Ensembl\|ENSACAG00000011789\|\|G1KLI5 | CHRNA9 | Uncharacterized protein | ligand-gated ion channel | neuron projection (GO:0043005) |
| NVE2635 | HUMAN\|HGNC\|1463\|\|Q13555 | KCC2G | Calcium/calmodulin-dependent protein kinase type II subunit gamma | non-receptor serine/threonine protein kinase | neuron projection (GO:0043005) |
| NVE5017 | HUMAN\|HGNC\|1396\|\|Q9P0X4 | CAC1I | Voltage-dependent T-type calcium channel subunit alpha-1I | voltage-gated ion channel | neuron projection (GO:0043005) |
| NVE6180 | RAT\|RGD\|2348\|\|Q05941 | ACHA7 | Neuronal acetylcholine receptor subunit alpha-7 | ligand-gated ion channel | neuron projection (GO:0043005) |
| NVE13547 | MOUSE\|MGI\|MGI\|2441950\|\|Q80TS3 | AGRL3 | Adhesion G protein-coupled receptor L3 | G-protein coupled receptor | transmembrane signal receptor (PC00197) |
| NVE14451 | CHICK\|Ensembl\|ENSGALG00000029392\|\|P19019 | GBRB3 | Gamma-aminobutyric acid receptor subunit beta-3 | ligand-gated ion channel | neuron projection (GO:0043005) |
| NVE1484 | DROME\|FlyBase\|FBgn0004622\|\|P30975 | TLR2 | Tachykinin-like peptides receptor 99D | G-protein coupled receptor | transmembrane signal receptor (PC00197) |
| NVE7617 | HUMAN\|HGNC\|10588\|\|Q99250 | NAC2 | Sodium channel protein type 2 subunit alpha | voltage-gated ion channel | neuron projection (GO:0043005) |
| NVE12387 | BOVIN\|Gene\|CAMK2D\|\|Q2HJF7 | KCC2D | Calcium/calmodulin-dependent protein kinase type II subunit delta | non-receptor serine/threonine protein kinase | neuron projection (GO:0043005) |
| NVE13402 | CHICK\|Ensembl\|ENSGALG00000004446\|\|Q75XU5 | NPFF1 | Neuropeptide FF receptor 1 | G-protein coupled receptor | transmembrane signal receptor (PC00197) |
| NVE23178 | DROME\|FlyBase\|FBgn0004168\|\|P28285 | 5HT2A | 5-hydroxytryptamine receptor 2A | G-protein coupled receptor | somatodendritic compartment (GO:0036477) |
| NVE25668 | DROME\|FlyBase\|FBgn0000036\|\|P09478 | ACH1 | Acetylcholine receptor subunit alpha-like 1 | ligand-gated ion channel | neuron projection (GO:0043005) |
| NVE22703 | DANRE\|ZFIN\|ZDB-GENE-040801-92\|\|Q6DC77 | MPRGB | Membrane progestin receptor gamma-B | G-protein coupled receptor | transmembrane signal receptor (PC00197) |
| NVE24524 | HUMAN\|HGNC\|11018\|\|Q9H598 | VIAAT | Vesicular inhibitory amino acid transporter | - | neuron projection (GO:0043005) |
| NVE7386 | DROME\|FlyBase\|FBgn0038880\|\|Q8IN35 | SIFAR | Neuropeptide SIFamide receptor | G-protein coupled receptor | transmembrane signal receptor (PC00197) |
| NVE21442 | HUMAN\|HGNC\|15781\|\|Q494W8 | CRFM7 | CHRNA7-FAM7A fusion protein | ligand-gated ion channel | neuron projection (GO:0043005) |
| NVE20517 | MOUSE\|MGI\|MGI\|1860130\|\|Q924H0 | NPFF2 | Neuropeptide FF receptor 2 | G-protein coupled receptor | transmembrane signal receptor (PC00197) |
| NVE2517 | DANRE\|ZFIN\|ZDB-GENE-070705-309\|\|A0A0R4IVA4 | CC14A | Dual specificity protein phosphatase CDC14AB | - | neuron projection (GO:0043005) |
| NVE8357 | HUMAN\|HGNC\|5299\|\|Q13639 | 5HT4R | 5-hydroxytryptamine receptor 4 | G-protein coupled receptor | neuron projection (GO:0043005) |
| NVE23188 | ANOCA\|Ensembl\|ENSACAG00000014117\|\|G1KPI2 | SYT4 | Uncharacterized protein | membrane trafficking regulatory protein | neuron projection (GO:0043005) |
| NVE13933 | MOUSE\|MGI\|MGI\|2677838\|\|A2AJ76 | HMCN2 | Hemicentin-2 | transmembrane signal receptor | transmembrane signal receptor (PC00197) |
| NVE1327 | FELCA\|Gene\|OPN4\|\|Q5YKK9 | OPN4 | Melanopsin | G-protein coupled receptor | transmembrane signal receptor (PC00197) |
| NVE17221 | MOUSE\|MGI\|MGI\|104311\|\|P32240 | PE2R4 | Prostaglandin E2 receptor EP4 subtype | G-protein coupled receptor | transmembrane signal receptor (PC00197) |
| NVE10027 | CHICK\|Ensembl\|ENSGALG00000049848\|\|A0A3Q2TXF0 | CNR1 | Cannabinoid receptor | G-protein coupled receptor | transmembrane signal receptor (PC00197) |
| NVE19228 | HUMAN\|HGNC\|5299\|\|Q13639 | 5HT4R | 5-hydroxytryptamine receptor 4 | G-protein coupled receptor | somatodendritic compartment (GO:0036477) |
| NVE15844 | MACMU\|Ensembl\|ENSMMUG00000020241\|\|Q9GK75 | NPY1R | Neuropeptide Y receptor Y1 | G-protein coupled receptor | transmembrane signal receptor (PC00197) |
| NVE17029 | MOUSE\|MGI\|MGI\|2429764\|\|P69744 | CAT2 | Transient receptor potential cation channel subfamily V member 5 | ion channel | neuron projection (GO:0043005) |
| NVE1501 | CANLF\|Ensembl\|ENSCAFG00000013828\|\|P11617 | AA2AR | Adenosine receptor A2a | G-protein coupled receptor | transmembrane signal receptor (PC00197) |
| NVE12996 | RAT\|RGD\|1308804\|\|Q5XIU9 | PGRC2 | Membrane-associated progesterone receptor component 2 | transmembrane signal receptor | transmembrane signal receptor (PC00197) |
| NVE26217 | FELCA\|Gene\|OPN4\|\|Q5YKK10 | OPN5 | Melanopsin | G-protein coupled receptor | transmembrane signal receptor (PC00197) |
| NVE6519 | CAEEL\|WormBase\|WBGene00016610\|\|Q94177 | ADRL | Progestin and adipoQ receptor-like protein 1 | G-protein coupled receptor | transmembrane signal receptor (PC00197) |
| NVE10190 | DROME\|FlyBase\|FBgn0038980\|\|Q9VCZ3 | OCTB1 | Octopamine receptor beta-1R | G-protein coupled receptor | transmembrane signal receptor (PC00197) |
| NVE21472 | NEMVE\|EnsemblGenome\|NEMVEDRAFT_v1g248535\|\|A7T1N0 | TMP2L | Transient receptor potential cation channel subfamily M member-like 2 | ion channel | neuron projection (GO:0043005) |
| NVE24515 | MOUSE\|MGI\|MGI\|892032\|\|P97401 | FRITZ | Secreted frizzled-related protein 3 | transmembrane signal receptor | transmembrane signal receptor (PC00197) |
| NVE25785 | MOUSE\|MGI\|MGI\|99456\|\|Q01727 | MSHR | Melanocyte-stimulating hormone receptor | G-protein coupled receptor | transmembrane signal receptor (PC00197) |
| NVE6925 | HUMAN\|HGNC\|10585\|\|P35498 | NAC1 | Sodium channel protein type 1 subunit alpha | voltage-gated ion channel | neuron projection (GO:0043005) |
| NVE14868 | MOUSE\|MGI\|MGI\|2664668\|\|Q7TN88 | PK1L2 | Polycystic kidney disease protein 1-like 2 | ion channel | neuron projection (GO:0043005) |
| NVE17821 | ANOCA\|Ensembl\|ENSACAG00000005650\|\|G1KEG9 | GLRA3 | Uncharacterized protein | ligand-gated ion channel | neuron projection (GO:0043005) |
| NVE12212 | BOVIN\|Ensembl\|ENSBTAG00000003446\|\|E1BIE4 | EPHB4 | EPH receptor B4 | - | neuron projection (GO:0043005) |
| NVE7681 | BOVIN\|Gene\|HMCN1\|\|E1BE11 | HMCN1 | Uncharacterized protein | tyrosine protein kinase receptor | transmembrane signal receptor (PC00197) |
| NVE24164 | BOVIN\|Gene\|HMCN1\|\|E1BE11 | HMCN1 | Uncharacterized protein | tyrosine protein kinase receptor | transmembrane signal receptor (PC00197) |
| NVE25358 | HUMAN\|HGNC\|11119\|\|Q99835 | SMO | Smoothened homolog | transmembrane signal receptor | neuron projection (GO:0043005) |
| NVE19422 | DROME\|FlyBase\|FBgn0086906\|\|Q9I7U4 | TITIN | Titin | tyrosine protein kinase receptor | transmembrane signal receptor (PC00197) |
| NVE3573 | MOUSE\|MGI\|MGI\|97175\|\|P20357 | MTAP2 | Microtubule-associated protein 2 | - | neuron projection (GO:0043005) |
| NVE21871 | MOUSE\|MGI\|MGI\|1196627\|\|Q9R1C8 | 5HT6R | 5-hydroxytryptamine receptor 6 | G-protein coupled receptor | neuron projection (GO:0043005) |
| NVE2492 | DANRE\|Ensembl\|ENSDARG00000013649\|\|F1R4B10 | CASR | Calcium-sensing receptor | G-protein coupled receptor | transmembrane signal receptor (PC00197) |
| NVE16130 | CHICK\|Gene\|FSHR\|\|P79763 | FSHR | Follicle-stimulating hormone receptor | G-protein coupled receptor | transmembrane signal receptor (PC00197) |
| NVE11916 | HUMAN\|HGNC\|21293\|\|Q8NDA2 | HMCN2 | Hemicentin-2 | transmembrane signal receptor | transmembrane signal receptor (PC00197) |
| NVE19915 | CHICK\|Ensembl\|ENSGALG00000002118\|\|O93393 | CO6 | Calcium-activated potassium channel beta subunit | voltage-gated ion channel | neuron projection (GO:0043005) |
| NVE13710 | CAEEL\|WormBase\|T23D8.1b\|\|A0A0K3AWM6 | MOM5 | Protein mom-5 | transmembrane signal receptor | transmembrane signal receptor (PC00197) |
| NVE19835 | DROME\|FlyBase\|FBgn0038880\|\|Q8IN35 | SIFAR | Neuropeptide SIFamide receptor | G-protein coupled receptor | transmembrane signal receptor (PC00197) |
| NVE17972 | DANRE\|ZFIN\|ZDB-GENE-990714-19\|\|Q9W7R4 | TENM3 | Teneurin-3 | - | neuron projection (GO:0043005) |
| NVE2156 | CHICK\|Gene\|CHRNA10\|\|Q9I8C7 | ACH10 | Neuronal acetylcholine receptor subunit alpha-10 | ligand-gated ion channel | neuron projection (GO:0043005) |
| NVE20802 | DANRE\|ZFIN\|ZDB-GENE-080221-1\|\|F1Q681 | NPY1R | Neuropeptide Y receptor Y1 | G-protein coupled receptor | transmembrane signal receptor (PC00197) |
| NVE8662 | HUMAN\|HGNC\|11049\|\|Q01959 | SC6A3 | Sodium-dependent dopamine transporter | primary active transporter | neuron projection (GO:0043005) |
| NVE19257 | RAT\|RGD\|1564772\|\|F1M4Q3 | HMCN1 | Hemicentin 1 | - | neuron projection (GO:0043005) |
| NVE275 | RAT\|RGD\|621570\|\|Q9EP86 | NPFF1 | Neuropeptide FF receptor 1 | G-protein coupled receptor | transmembrane signal receptor (PC00197) |
| NVE9473 | DROME\|FlyBase\|FBgn0004842\|\|P25931 | RYAR | RYamide receptor | G-protein coupled receptor | transmembrane signal receptor (PC00197) |
| NVE4599 | DROME\|FlyBase\|FBgn0004841\|\|P30974 | TLR1 | Tachykinin-like peptides receptor 86C | G-protein coupled receptor | transmembrane signal receptor (PC00197) |
| NVE12144 | HUMAN\|HGNC\|14079\|\|Q9UGM1 | ACHA9 | Neuronal acetylcholine receptor subunit alpha-9 | ligand-gated ion channel | neuron projection (GO:0043005) |
| NVE14952 | RAT\|RGD\|1564772\|\|F1M4Q3 | HMCN1 | Hemicentin 1 | - | neuron projection (GO:0043005) |
| NVE8663 | MOUSE\|MGI\|MGI\|96285\|\|Q60857 | SC6A4 | Sodium-dependent serotonin transporter | primary active transporter | neuron projection (GO:0043005) |
| NVE19358 | DANRE\|ZFIN\|ZDB-GENE-991117-1\|\|O93430 | GLRA1 | Glycine receptor subunit alphaZ1 | ligand-gated ion channel | neuron projection (GO:0043005) |
| NVE24392 | HUMAN\|HGNC\|9967\|\|P07949 | RET | Proto-oncogene tyrosine-protein kinase receptor Ret | - | neuron projection (GO:0043005) |
| NVE4207 | CHICK\|Ensembl\|ENSGALG00000042626\|\|A0A1D5PCG4 | SYT4 | Uncharacterized protein | membrane trafficking regulatory protein | neuron projection (GO:0043005) |
| NVE9028 | DROME\|FlyBase\|FBgn0034476\|\|Q7KIN0 | TOLL7 | Toll-like receptor 7 | transmembrane signal receptor | transmembrane signal receptor (PC00197) |
| NVE1388 | CHICK\|Gene\|MC1R\|\|P55167 | MSHR | Melanocyte-stimulating hormone receptor | G-protein coupled receptor | transmembrane signal receptor (PC00197) |
| NVE25364 | RAT\|RGD\|1564772\|\|F1M4Q3 | HMCN1 | Hemicentin 1 | - | neuron projection (GO:0043005) |
| NVE21855 | MOUSE\|MGI\|MGI\|98328\|\|P30875 | SSR2 | Somatostatin receptor type 2 | G-protein coupled receptor | neuron projection (GO:0043005) |
| NVE21136 | DANRE\|Ensembl\|ENSDARG00000013649\|\|F1R4B8 | CASR | Calcium-sensing receptor | G-protein coupled receptor | transmembrane signal receptor (PC00197) |
| NVE23050 | RAT\|RGD\|1564772\|\|F1M4Q3 | HMCN1 | Hemicentin 1 | - | neuron projection (GO:0043005) |
| NVE20106 | DANRE\|Ensembl\|ENSDARG00000013649\|\|F1R4B9 | CASR | Calcium-sensing receptor | G-protein coupled receptor | transmembrane signal receptor (PC00197) |
| NVE2371 | CHICK\|Ensembl\|ENSGALG00000002118\|\|O93393 | CO6 | Calcium-activated potassium channel beta subunit | voltage-gated ion channel | neuron projection (GO:0043005) |
| NVE7629 | DROME\|FlyBase\|FBgn0004622\|\|P30975 | TLR2 | Tachykinin-like peptides receptor 99D | G-protein coupled receptor | transmembrane signal receptor (PC00197) |
| NVE141 | MOUSE\|MGI\|MGI\|3041203\|\|Q80T32 | AGRD1 | Adhesion G-protein coupled receptor D1 | G-protein coupled receptor | transmembrane signal receptor (PC00197) |
| NVE11735 | MOUSE\|MGI\|MGI\|95749\|\|Q91XP5 | GLRA3 | Glycine receptor subunit alpha-3 | ligand-gated ion channel | neuron projection (GO:0043005) |
| NVE2876 | RAT\|RGD\|628837\|\|P60711 | ACTB | Actin, cytoplasmic 1 | actin and actin related protein | neuron projection (GO:0043005) |
| NVE25008 | CHICK\|Ensembl\|ENSGALG00000006130\|\|O93388 | NPC2 | Stathmin-3 | - | neuron projection (GO:0043005) |
| NVE4080 | RAT\|RGD\|1564772\|\|F1M4Q3 | HMCN1 | Hemicentin 1 | - | neuron projection (GO:0043005) |
| NVE5966 | MOUSE\|MGI\|MGI\|95749\|\|Q91XP5 | GLRA3 | Glycine receptor subunit alpha-3 | ligand-gated ion channel | neuron projection (GO:0043005) |
| NVE9440 | RAT\|RGD\|1305841\|\|D4A6L0 | GP158 | Probable G-protein coupled receptor 158 | G-protein coupled receptor | transmembrane signal receptor (PC00197) |
| NVE18296 | MOUSE\|MGI\|MGI\|97281\|\|P13595 | NCAM1 | Neural cell adhesion molecule 1 | - | neuron projection (GO:0043005) |
| NVE17393 | MOUSE\|MGI\|MGI\|1347520\|\|Q9Z139 | ROR1 | Inactive tyrosine-protein kinase transmembrane receptor ROR1 | - | neuron projection (GO:0043005) |
| NVE9727 | MOUSE\|MGI\|MGI\|99402\|\|Q60613 | AA2AR | Adenosine receptor A2a | G-protein coupled receptor | transmembrane signal receptor (PC00197) |
| NVE20788 | HUMAN\|HGNC\|3039\|\|O60469 | DSCAM | Down syndrome cell adhesion molecule | scaffold/adaptor protein | neuron projection (GO:0043005) |
| NVE6463 | DROME\|FlyBase\|FBgn0038880\|\|Q8IN36 | SIFAR | Neuropeptide SIFamide receptor | G-protein coupled receptor | transmembrane signal receptor (PC00197) |
| NVE13212 | RAT\|RGD\|2353\|\|P09660 | ACHE | Acetylcholine receptor subunit epsilon | ligand-gated ion channel | neuron projection (GO:0043005) |
| NVE26151 | MOUSE\|MGI\|MGI\|1347095\|\|Q9Z0M6 | CD97 | CD97 antigen | G-protein coupled receptor | transmembrane signal receptor (PC00197) |
| NVE6241 | HUMAN\|HGNC\|11333\|\|P31391 | SSR4 | Somatostatin receptor type 4 | G-protein coupled receptor | neuron projection (GO:0043005) |
| NVE7440 | HUMAN\|HGNC\|18992\|\|Q8IZF6 | AGRG4 | Adhesion G-protein coupled receptor G4 | G-protein coupled receptor | transmembrane signal receptor (PC00197) |
| NVE8550 | RAT\|RGD\|619891\|\|Q8VHD7 | GPR83 | G protein-coupled receptor 83 | G-protein coupled receptor | transmembrane signal receptor (PC00197) |
| NVE2244 | CHICK\|Ensembl\|ENSGALG00000015126\|\|A0A1D5PAY3 | TRPM3 | Uncharacterized protein | ion channel | neuron projection (GO:0043005) |
| NVE3729 | HUMAN\|HGNC\|10450\|\|O14718 | OPSX | Visual pigment-like receptor peropsin | G-protein coupled receptor | neuron projection (GO:0043005) |
| NVE12143 | RAT\|RGD\|1564772\|\|F1M4Q3 | HMCN1 | Hemicentin 1 | - | neuron projection (GO:0043005) |
| NVE16819 | DANRE\|ZFIN\|ZDB-GENE-050913-90\|\|A0T2N3 | APJB | Apelin receptor B | G-protein coupled receptor | transmembrane signal receptor (PC00197) |
| NVE12516 | MOUSE\|MGI\|MGI\|1333877\|\|O88495 | MTR1L | Melatonin-related receptor | G-protein coupled receptor | transmembrane signal receptor (PC00197) |
| NVE13879 | DANRE\|ZFIN\|ZDB-GENE-060427-5\|\|E7FCE4 | ROR2 | Receptor tyrosine kinase-like orphan receptor 2 | - | neuron projection (GO:0043005) |
| NVE24406 | HUMAN\|HGNC\|21293\|\|Q8NDA2 | HMCN2 | Hemicentin-2 | transmembrane signal receptor | transmembrane signal receptor (PC00197) |
| NVE19889 | HUMAN\|HGNC\|4600\|\|O00222 | GRM8 | Metabotropic glutamate receptor 8 | G-protein coupled receptor | transmembrane signal receptor (PC00197) |
| NVE16011 | MOUSE\|MGI\|MGI\|104295\|\|Q60898 | SLAP1 | Src-like-adapter | - | neuron projection (GO:0043005) |
| NVE21548 | MOUSE\|MGI\|MGI\|99779\|\|P49582 | ACHA7 | Neuronal acetylcholine receptor subunit alpha-7 | ligand-gated ion channel | neuron projection (GO:0043005) |
| NVE10021 | RAT\|RGD\|1564772\|\|F1M4Q3 | HMCN1 | Hemicentin 1 | - | neuron projection (GO:0043005) |
| NVE2362 | CHICK\|Ensembl\|ENSGALG00000002118\|\|O93393 | CO6 | Calcium-activated potassium channel beta subunit | voltage-gated ion channel | neuron projection (GO:0043005) |
| NVE1835 | HUMAN\|HGNC\|4039\|\|Q9ULW2 | FZD10 | Frizzled-10 | transmembrane signal receptor | transmembrane signal receptor (PC00197) |
| NVE15043 | DANRE\|ZFIN\|ZDB-GENE-070705-262\|\|A5WWA0 | ZNRF3 | E3 ubiquitin-protein ligase znrf3 | transmembrane signal receptor | transmembrane signal receptor (PC00197) |
| NVE5945 | RAT\|RGD\|1564772\|\|F1M4Q3 | HMCN1 | Hemicentin 1 | - | neuron projection (GO:0043005) |
| NVE7065 | RAT\|RGD\|1564772\|\|F1M4Q3 | HMCN1 | Hemicentin 1 | - | neuron projection (GO:0043005) |

**Fig. S1. Additional neuronal associated genes DE between the apical and oral regions.** Heatmap shows the relative expression of neuronal associated genes among oral or apical region of *Nematostella* larvae. The gene id and additional details are shown on the right.

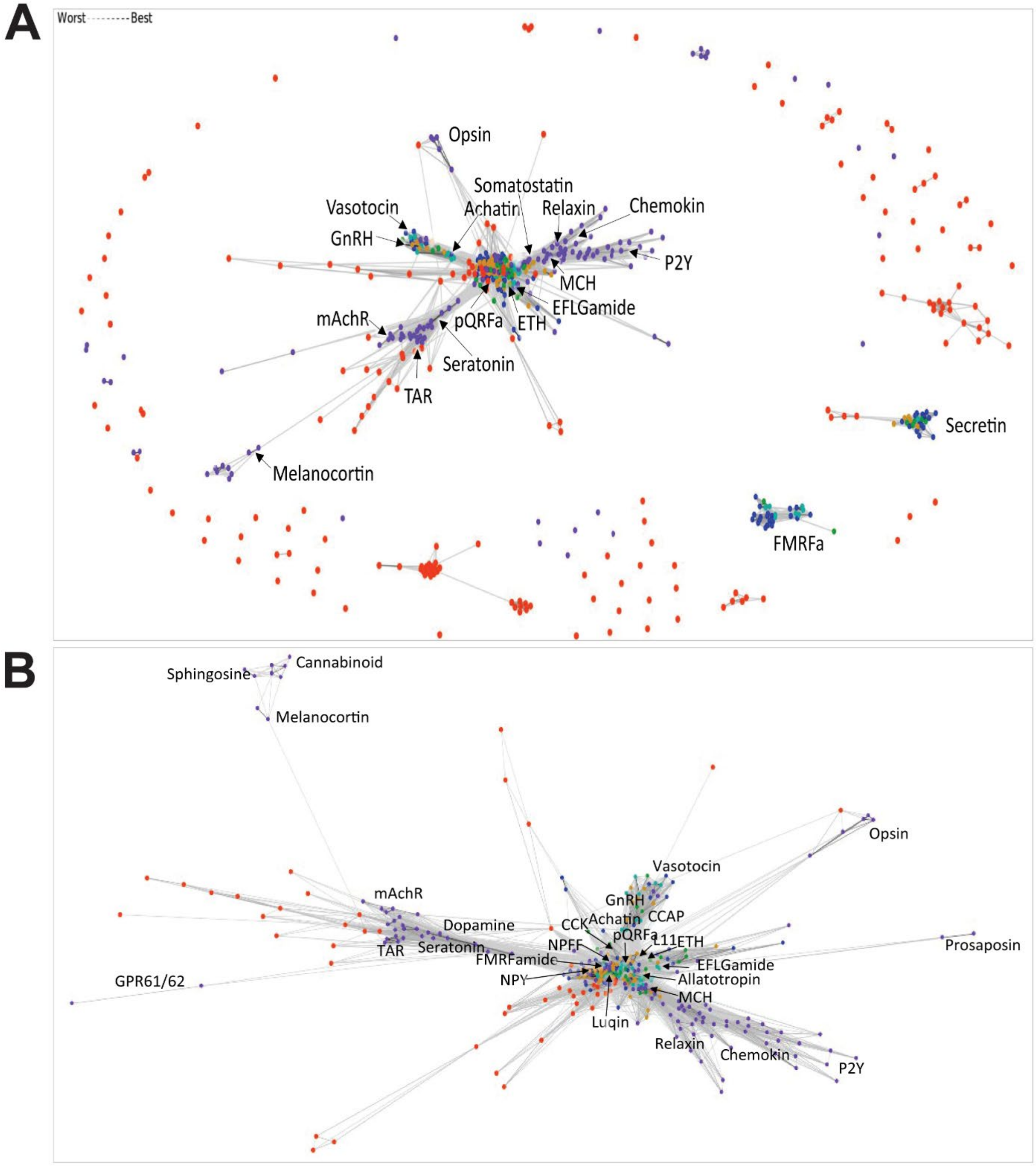


**Fig.S2. (A)** Sequence-similarity-based clustering of all *Nematostella* apical enriched GPCRs. Forty-six *Nematostella* GPCRs clustered with a large group of Rhodopsin and four clustered with Secretin. **(B)** Cluster map of the Rhodopsin cluster presented in A.

Development • Supplementary information

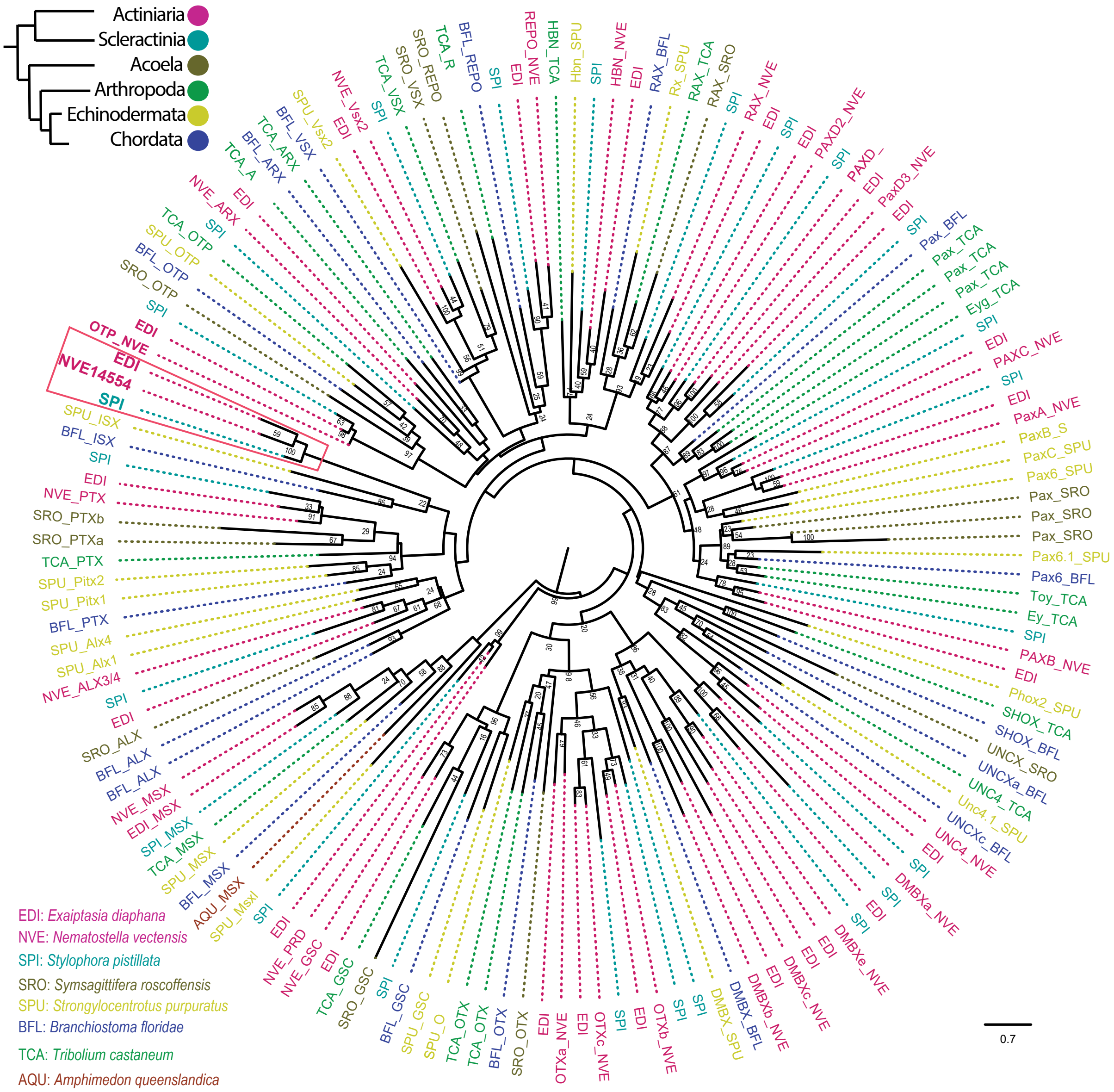


**Fig. S3. Maximum likelihood phylogenetic tree of PRD class homeobox of selected animals.** The homeobox sequences of a representative selection of Cnidarians and bilaterian lineages **(Supplementary Table 1)** were aligned with MUSCLE algorithm [77] in the SeaView program [78]. The SMS: Smart Model Selection in PhyML [79] was used to determine the best-fitting model for the Phylogenic reconstruction VT model (+G+F). The maximum-likelihood (ML) phylogenetic trees were constructed using PhyML v3.0 with 100 bootstrap [76]. Only bootstrap values of at least 20 at nodes are shown. The MSX family of ANTP class homeobox was used as an outgroup.

Development • Supplementary information

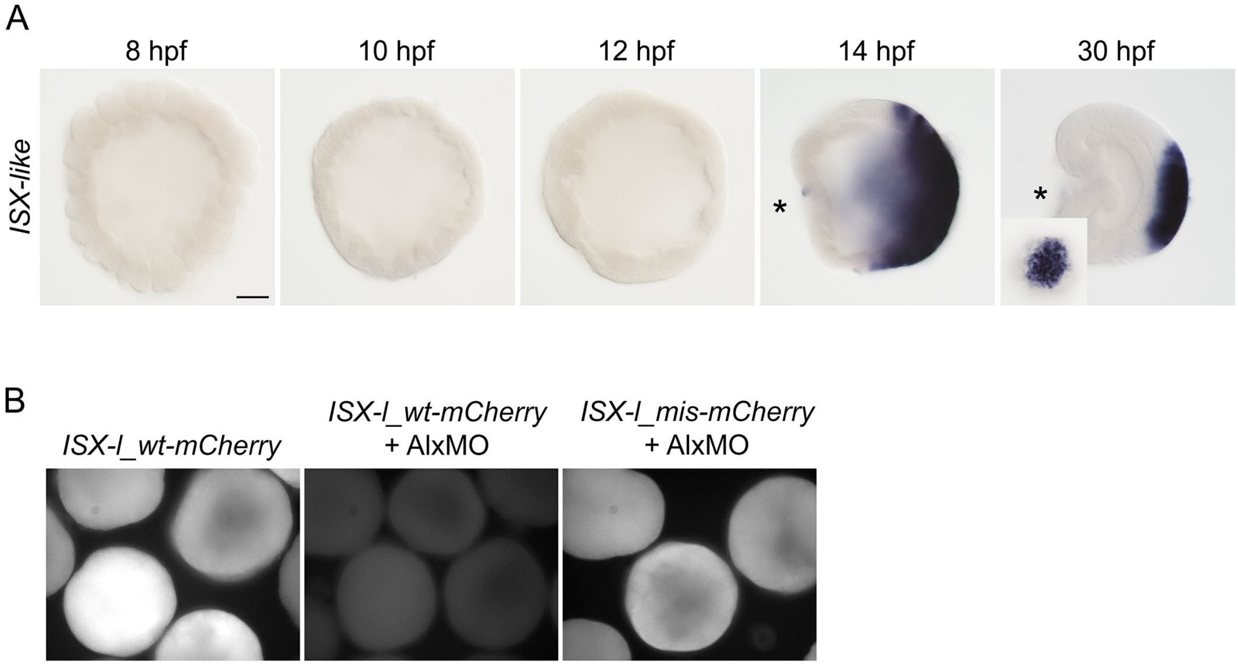


**Fig. S4. RNAi and morpholino efficiency. (A)** Early expression of ISX-like in *Nematostella* embryos. Inset on the 30 hpf gastrula image shows the aboral view. **(B)** Translation of the *mCherry* mRNA carrying the wild type recognition site of the ISX-like MO fused to it is efficiently suppressed by co-injecting ISX-like MO. In contrast, *mCherry* mRNA carrying the recognition site of the ISX-like MO with five introduced mismatches is efficiently translated in the presence of ISX-like MO. Lateral and aboral views, on lateral views, asterisk denotes the oral end. Scale bars 50 µm.

Development • Supplementary information

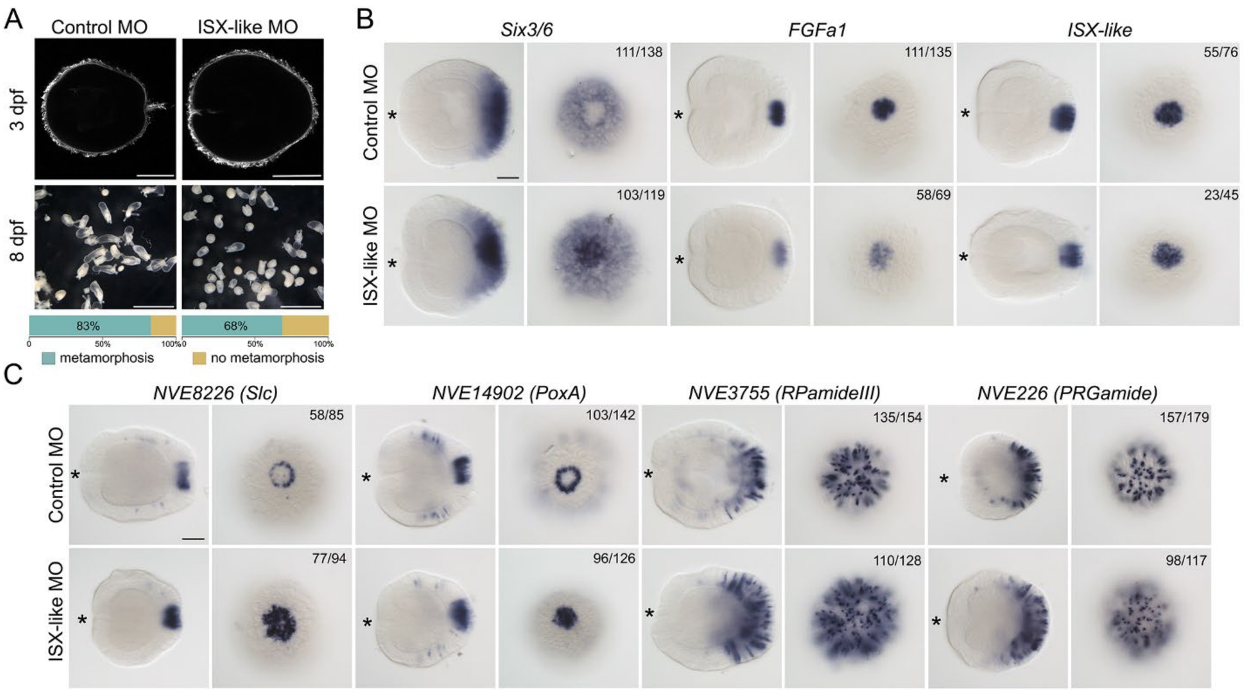


**Fig. S5. Effect of the morpholino-mediated knockdown of *ISX-like* phenocopies *ISX-like* RNAi. (A)** Morpholino knockdown of *ISX-like* leads to a loss of the apical tuft (anti-acetylated tubulin staining, top row) and a mild reduction in the metamorphosis rate. **(B)** In ISX-like morphants, Six3/6 expression expands into the apical domain, however, *FGFa1* and *ISX-like* are not strongly affected. **(C)** In ISX-like morphants, the expression of the ring genes extends into the pot domain, but the expression of the aborally enriched *PRGamide* (*NVE226*), and *RPamide III* (*NVE3775*) does not appear to be strongly affected. Lateral and aboral views, on lateral views, asterisk denotes the oral end. Scale bars on **(A)** 100 µm, on **(B)** and **(C)** 50 µm.

Development • Supplementary information

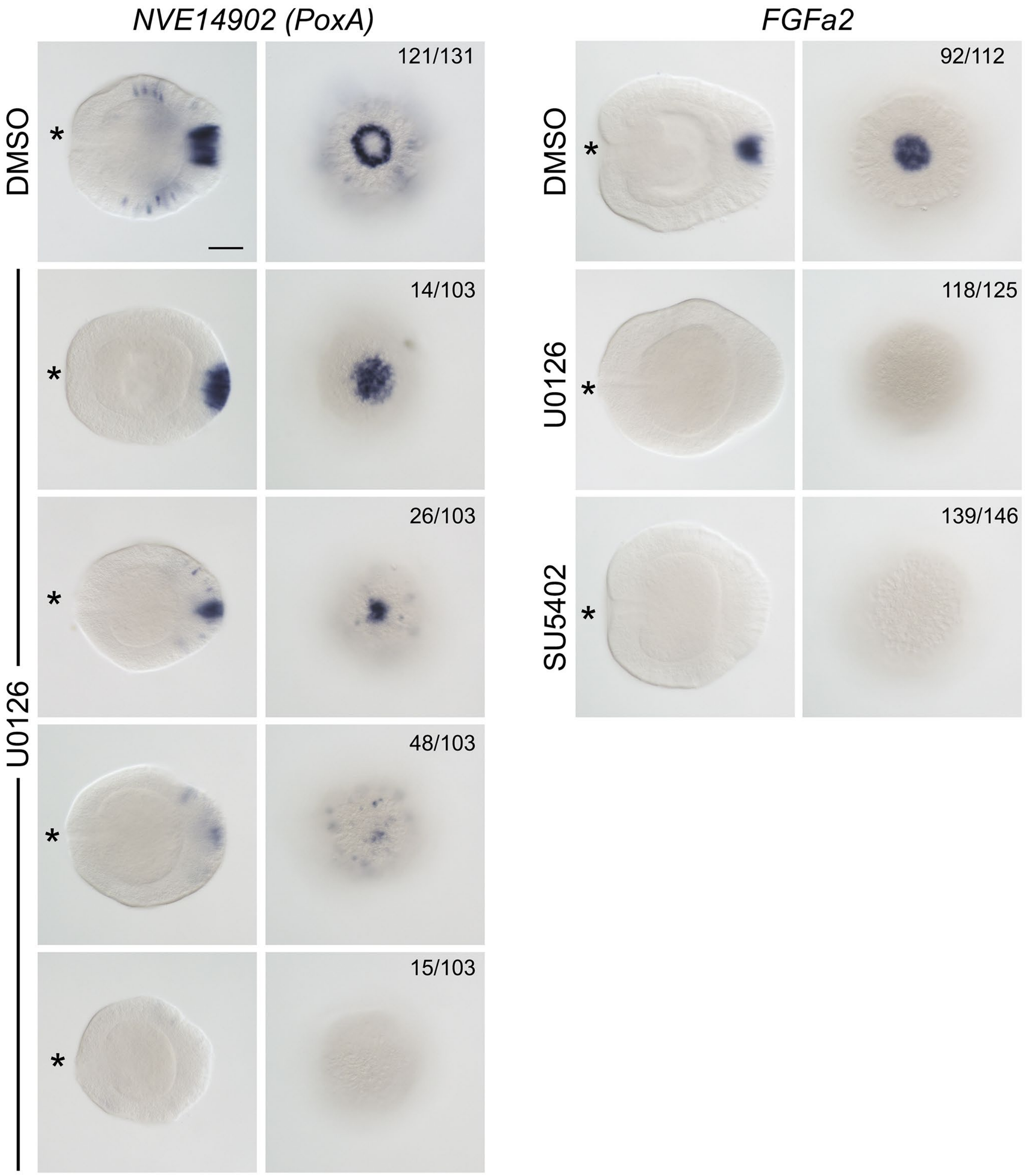


**Fig. S6. Variability of the effect of the U0126 treatment on NVE14902 expression and the effect of U0126 and SU5402 on FGFa2 expression.** Scale bar 50 µm.

**Table S1.** Data relevant to figures 2, 3, 4, S1, S2 and S3.

Click here to download Table S1

Development • Supplementary information

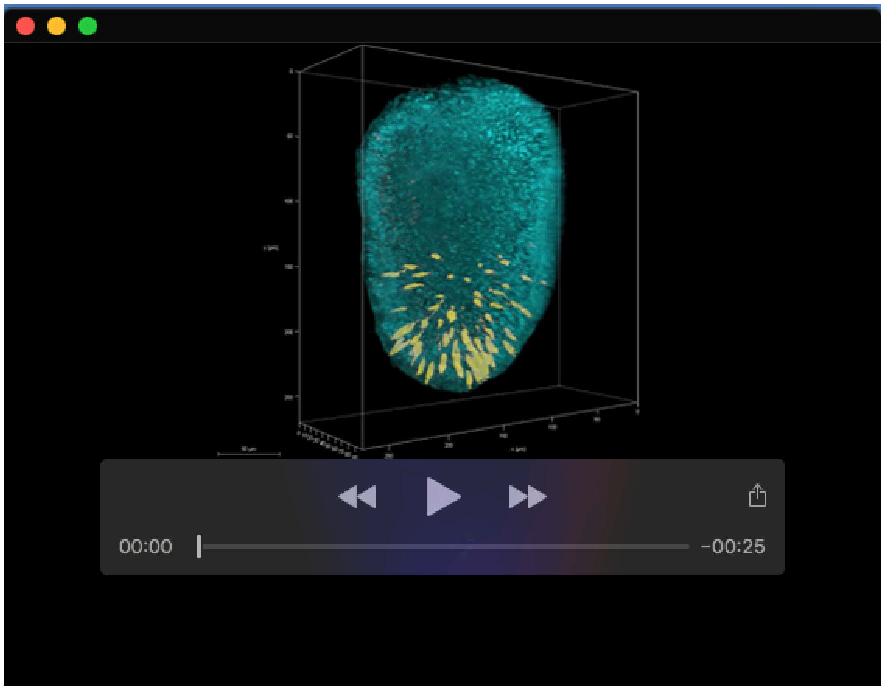


**Movie 1.** 3D view of fluorescence in situ hybridisation for gland/secretory cells type 1, related to Figure 5B&C. gland/secretory cells type 1 marker NVE22589 (yellow) and DAPI in cyan.

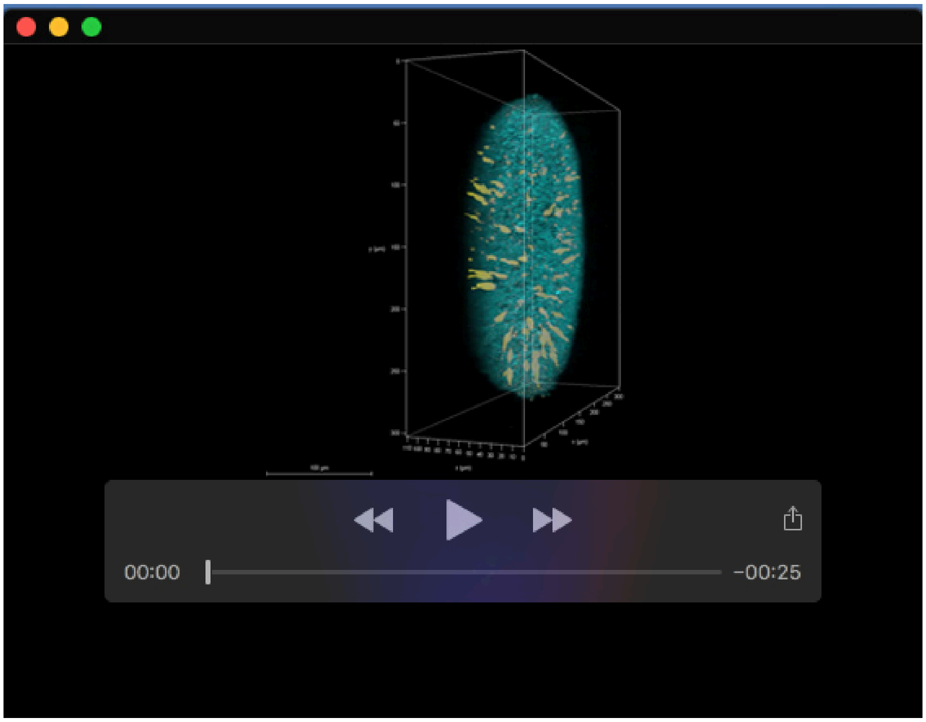


**Movie 2.** 3D view of fluorescence in situ hybridisation for gland/secretory cells type 3, related to Figure 5F. Gland/secretory cells type 3 marker NVE23674 (yellow) and DAPI in cyan.

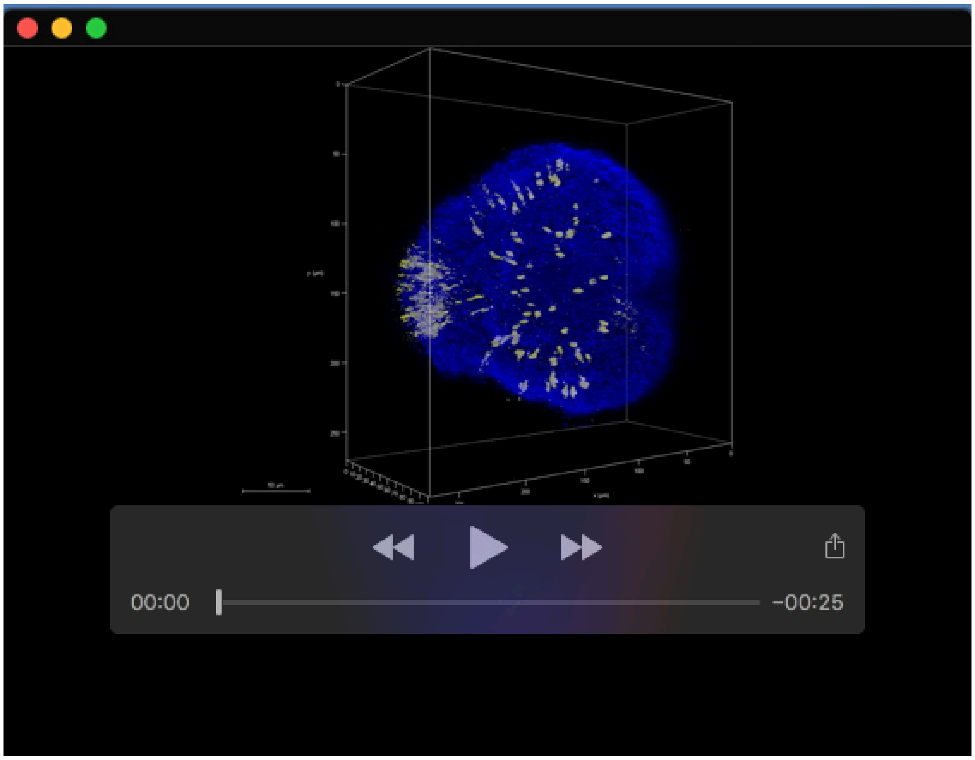


**Movie 3.** 3D view of fluorescence in situ hybridisation for larval-specific neuron gene, related to Figure 6A. *NVE8226* mRNA probe (yellow) and DAPI in Blue.

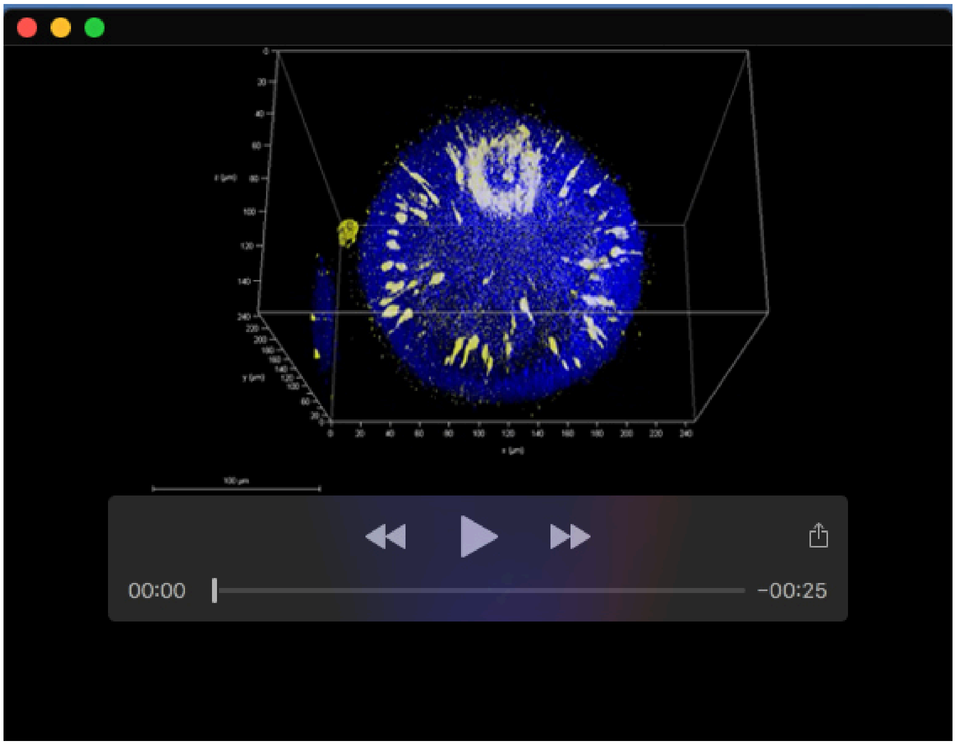


**Movie 4.** 3D view of fluorescence in situ hybridisation for larval-specific neuron gene, related to Figure 6E. *NVE14902* mRNA probe (yellow) and DAPI in Blue.

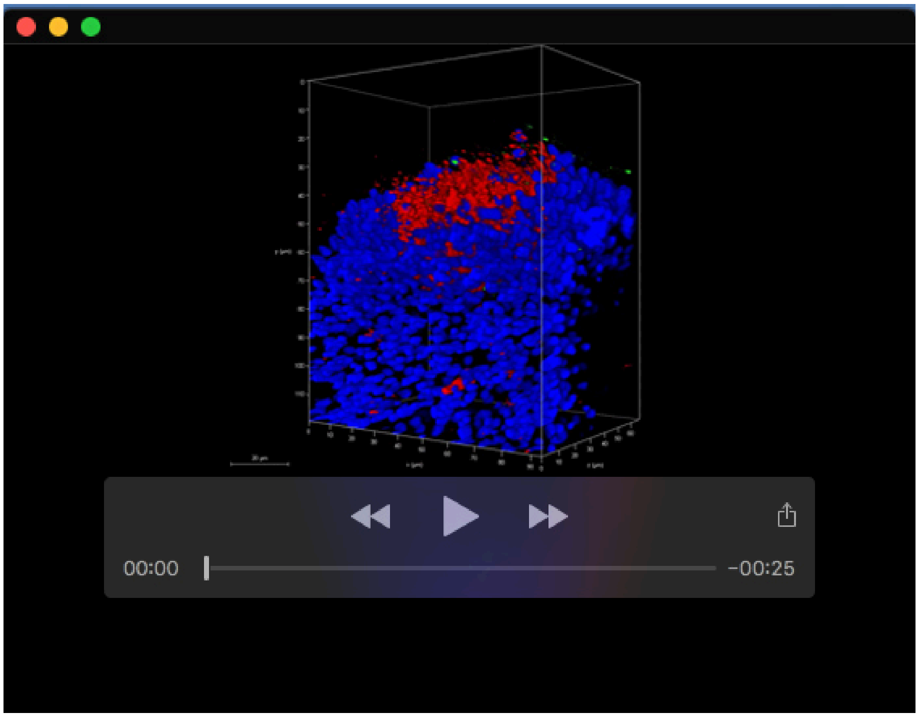


**Movie 5.** 3D view of Double fluorescence in situ hybridisation for spot probe *NVE14554* (green) and rind NVE8226 (red) mRNA probes, related to Figure 6L. DAPI in Blue.

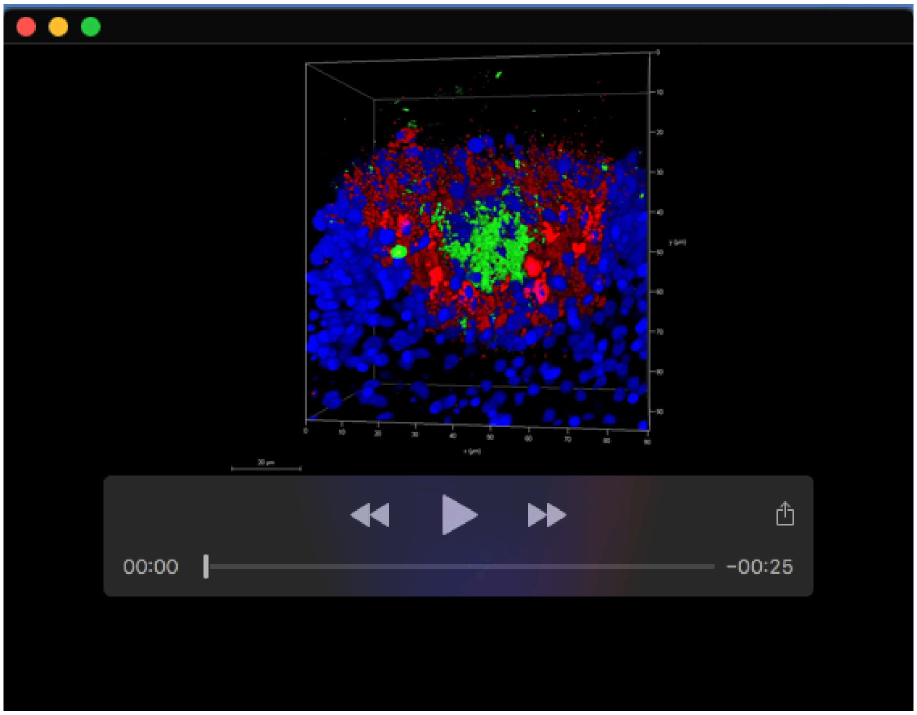


**Movie 6.** 3D view of Double fluorescence in situ hybridisation for spot probe *NVE14554* (green) and rind *NVE14902* (red) mRNA probes, related to Figure 6M. DAPI in Blue.
