# Supplementary material for: Molecular and cellular architecture of the larval sensory organ in the cnidarian Nematostella vectensis
Source: Development. 2022 Aug 24;149(16):dev200833. doi: 10.1242/dev.200833 (PMC9481973; doi:10.1242/dev.200833)
Supplement: Reviewer comments [file develop-149-200833_review_history.pdf]

Molecular and cellular architecture of the larval sensory organ in the cnidarian *Nematostella vectensis*

Eleanor Gilbert, Callum Teeling, Tatiana Lebedeva, Siffreya Pedersen, Nathan Chrismas, Grigory Genikhovich and Vengamanaidu Modepalli
DOI: 10.1242/dev.200833

Editor: Cassandra Extavour

Review timeline

Original submission:	10 May 2021
Editorial decision:	5 Jul 2021
Resubmission:	12 April 2022
Editorial decision:	23 May 2022
First revision received:	4 July 2022
Accepted:	18 July 2022

Original submission

First decision letter

MS ID#: DEVELOP/2021/199789

MS TITLE: Molecular and cellular architecture of the larval sensory organ in the cnidarian Nematostella vectensis

AUTHORS: Callum Teeling, Eleanor Gilbert, Siffreya Pedersen, Nathan Chrismas, and Vengamanaidu Modepalli

Dear Dr. Modepalli,

I have now received all the referees' reports on the above manuscript, and have reached a decision. I am sorry to say that the outcome is not a positive one. The referees' comments are appended below, or you can access them online: please go to Development's submission site and click on the 'Manuscripts with Decisions' queue in the Author Area.

As you will see, the referees raise some significant concerns about your paper, and are not strongly in favour of publication. Having looked at the manuscript myself, I agree with their views, and I must therefore, reject your paper. The main issues with this manuscript are the lack of follow through in the discussion in interpreting these data as advancing novel conclusions about the evolution of these organs, and the lack of functional experiments to validate the putative roles of any of the identified genes in organ function. In principle if such changes were made to the manuscript it might be suitable for consideration for publication in Development (following peer review), but these changes are so significant that I believe they are beyond the scope of a major revision.

Yours sincerely

Cassandra Extavour
Handling Editor
Development

© 2022. Published by The Company of Biologists under the terms of the Creative Commons Attribution License (https://creativecommons.org/licenses/by/4.0/).

Reviewer 1

Advance Summary and Potential Significance to Field

Molecular and cellular architecture of the larval sensory organ in the cnidarian Nematostella vectensis

Teeling et al.,

Summary: An apical organ is present in many marine invertebrate larvae, and is thought to be a sensory structure of the local environment. The organ has neuronal characteristics, but the resident cells have not been characterized in much depth. Although many studies have been conducted to identify its function and composition, it remains poorly understood, especially for such an important and prevalent structure in larvae, and for evolutionary studies. The present work integrates several new and used datasets to identify candidates of this organ. The investigators enrich for the apical organ by microdissection, and following RNA-seq of the apical region compared to the remaining larva, identifies differential gene expression to link to previously generated single cell RNA-seq datasets. The data appear to be significantly curated, and statistically analyzed in order to add to the list of genes expressed in the apical organ. Some of the RNA candidates are then tested by in situ hybridization, and those data are effectively collected, and appropriately interpreted. However, since the apical region dissected in this study is not an isolation, but is an enrichment, the statement and implications of "isolated apical organ" should be tempered. A recent in-depth scRNA-seq dataset was compiled, used here, and referenced appropriately. The integration of these new/previous data could be considered as redundant, but minimal larval analysis was accomplished in the previous study, and it lacked in situ hybridization analysis to validate many of the statements. The current study fills that gap with total focus on the larval apical organ. This work should add significantly to the understanding of the biology of the apical organ. Since the apical organ dissection was compared to the remaining larval cells, the apical/basal DEG analysis will add to better overall understanding of the larva. These mRNA were compared in depth and should be a strong resource for the future. Figure 2H has mis-spellings (Aborala, ubiquities) I found the end of the Results/Discussion section and the Conclusions section to be underperforming. They both do nothing more than reiterate the results or introduction and unfortunately, does not help the reader understand the significance and large picture of what this work is set up to do. I am concerned that a reader outside of the immediate subject will gain little in this absence, and especially when considering how highly touted the Apical Organ is in the Introduction for evolutionary reasons, the writing does not support the gravity of the results. The authors need dig much deeper to help the reader appreciate the impact this work should have. It does not.

Comments for the author

See above

Reviewer 2

Advance Summary and Potential Significance to Field

In this paper, Teeling et al further examine the cell identity of the apical region in Nematostella planula embryos. The study generated new transcriptomic data that are examined against previous single cell transcriptomic data (Sebe-Pedros et al 2018), whole body transcriptomic data from knockdown mutant embryos (Sinigaglia et al 2015) and whole body proteomic data (Sigg et al 2017). This data comparison was followed by in situ hybridization examination of some newly discovered genes being expressed in specific cell types including neurons and gland cells. The study provides spatial gene expression evidence that supports the proposition that the apical pole of Nematostella planula embryos may function as a larval sensory organ. Functional data via specific knock down / knock out analyses are still needed to further support this hypothesis. Nevertheless, this work complements previous studies and provides an updated list of apical genes that can be further studied in order to functionally dissect the role of the apical pole organ.

© 2022. Published by The Company of Biologists under the terms of the Creative Commons Attribution License (https://creativecommons.org/licenses/by/4.0/).

Comments for the author

General questions / comments:

1. There appears to be some discrepancy of embryonic data collection times among this paper and previous papers. This is especially important because these data are directly compared. Sebe - Pedros paper: Single cell data collected at 2d, 4d, 7d post fertilization (18.5C)
This paper: apical data collected at 50-60hpf, 75-85h post fertilization (18C)
Proteomic data from Sigg paper: collected at 3d post fertilization (18C)
How do the authors deal with this issue? How do they account for this difference when interpreting their data? This needs to be acknowledged and discussed.

2. How did the authors dissect the apical pole of these highly motile planulas? Were drugs used to immobilize them? Don't these drugs potentially affect the transcriptional readout? Discuss implications if drug was used.

3. Considering non-synchrony of development, were developmental landmarks used to sort out embryos among the early and late planula groups?

4. It will be informative to discuss how this study complements and enhances the single cell transcriptomic analysis approach (Sebe-Pedros 2018) that is widely used in recent developmental biology studies.

Specific comments / suggestions on figures:

1. Fig 1E should be 1D? 1D should be 1C? Not clear.
Define padj FDR
2. Fig 2 legend title should be more specific to what is presented
3. In Fig 3, images show late planula or early planula stages? Both? Indicate. Is the ring and spot correlated to dev stage? i.e. does the ring pattern become spot in a different dev stage? Or vice versa?
4. In Fig 4, what do the different groups Epithelium 1,2,3 etc refer to? How do these group definitions compare to the single cell data definitions from Sebe-Pedros et al paper?
5. In Fig 5 what is the stage shown? Early or late planula? Is there expected to be a different pattern between these stages?
Is there a difference between Fig. 5C and 5D? Legend description missing.
Embryo in 5J different to 5K stage-wise. Comment on the different expression pattern of ALX between these two stages. Is It consistent that the ALX territory appears narrower / wider in different stage embryos? Are images in Fig 5 max projections of slices? How many slices of what thickness? How are these embryos imaged differently so that the ring pattern shown in J/K is not obvious in A/E?
Are all images in Fig 5N important to show? Place the embryos with apical tuft to the left of the image to mirror cartoon for clarity. Was immuno-staining with acetyl-tubulin combined with in-situs to justify statement "The cells with apical tuft were concentrated in the apical pit visualised by immunostaining with an anti-tubulin antibody, where the spot genes like Alx are expressed"? Alx in situ combined with acetyl-tubulin staining will confirm this.
6. Better display of Fig 6 will be helpful. How is B different from C? Separate the apical from the body ISH of these gland cells with a top label to make it clearer. Labelled insets in J and O are confusing. Why are these images in insets? Keep consistency with insets only showing the apical view. Are all of these ISH essential to show here? Associate gland gene number NV....with Gland Group 1,2,3, 4 etc to tie the figure together. Also add the NV ID used in figure to the table shown in R. Explain color scheme in R, blue versus red. What are the numbers indicating?
7. In Fig 7 indicate color scheme used for in situs. Why do we see FISH for half of them? Combine E with F and G with H since they both show the same FISH but with different magnification. Indicate early or late planula stage of embryos shown.

Some typos in the text of figures should be corrected.

© 2022. Published by The Company of Biologists under the terms of the Creative Commons Attribution License (https://creativecommons.org/licenses/by/4.0/).

Reviewer 3

Advance Summary and Potential Significance to Field

see below

Comments for the author

Apical organs are ciliary sensory structures that are present at the top in many marine pelagic larvae. The fact that apical organs are present in various prebilaterian and bilateral groups (such as anthozoans, annelids, mollusks, and hemichordates) has made it a subject of extensive phylogenetic speculation about the origin of a centralized nervous system in metazoan evolution. Functionally, however, apical organs are not well characterized and, of course, apical organs could also have evolved convergently. For cnidarians it is likely that they act as chemo-/mechanosensory sensors in the attachment of larvae to suitable substrates and the subsequent induction of metamorphosis. - In the manuscript under review, the authors have generated the transcriptome of the apical organ in Nematostella and mapped it to existing sc RNAseq data (Sebe-Pedros et al 2018; Cell 173). In addition to neuronal and ciliated apical cells they identified gland cells and undifferentiated cells. The neuronal cells expressed neuropeptides specific for the aboral region (Nv-RPamide III and PRGamide) types. The expression profile of specific genes was validated by in situ hybridization. In addition, transcriptome profiling was performed by using previously published proteome data on cilia (Sigg et al. 2017; Developmental Cell 43), which revealed a subset of apical organ specific cilia proteins with likely non-motile functions. While the topic is interesting and nothing is wrong with the experiments (the double in situ data is excellent), the manuscript is lacking any functional analysis giving the reader a deeper insight into the development or function of the apical organ. As it is, it is a mere catalog of genes standing in the shadow of the previous work on developmental and neurobiology in Nematostella (Sebe-Pedros et al 2018; Cell 173; Sigg et al. 2017; Developmental Cell 43; Sinigaglia et al. 2015; Dev Biology 398).

Major points

Experimental approach. Sinigaglia et al (2015) already published a molecular characterization of the apical organ in Nematostella. They identified a set of genes (n=78) that was specific for the apical organ. To pick those genes they treated the animals with NvFGFa2 MO that was leading to larvae with an expanded apical organ. In the paper under review, the authors performed microdissection to separate the apical organ from the rest of the larval body of performed microdissection on Nematostella larvae to separate the apical organ from the rest of the larval body of the planulae; by this approach they identified about 15X more genes that were specific for the apical organ area (n=1185). However, even if the authors' transcriptome approach is much more sensitive, the Sinigaglia et al (2015) approach was much more specific because they were "picking" just the genes from the enlarged apical organ and not the entire larval background. I therefore I suggest that the authors complement their study by using planula exposed to an inhibited FGF signaling as done by Sinigaglia et al. This combined approach would provide a much more complete picture of the molecular and cellular architecture of the apical organ. By using early and late planulae, this approach would also place the cellular dynamics of the apical organ in a meaningful developmental and functional context.
Structure of the manuscript. The combination of results and discussion is often carried out in evo-devo studies describing large gene maps. However, this approach makes it very difficult for the reader to distinguish facts from interpretations. My suggestion is therefore to separate the two. The main text including the Introduction is also full of phylogenetic speculations, which is not helpful for a paper in the developmental context. Much of the discussion of a proposed homology of the larval brain structures across the phyla could be explained by convergent evolution, if functional data and not only comparative gene expression studies were available. My suggestion is therefore to radically shorten the Introduction, but also to thin out the "results" of phylogenetic speculations.

Specific points
In Figure 1, which gives a nice overview of the putative evolution of the apical organ, the authors show a deep split in Cnidarians between Medusozoa and Anthozoa, which is correct, but the nerve density in the "apical organ area" of Medusozoans is not correct. Medusozoa don't have a ciliary

© 2022. Published by The Company of Biologists under the terms of the Creative Commons Attribution License (https://creativecommons.org/licenses/by/4.0/).

tuft, but the neuron density at the aboral pole is also increased, there is no gap of RFamide or LW amide neurons at the aboral pole(see as an example: Seipp et al (2010) Neuronal cell death during metamorphosis of Hydractina echinata (Cnidaria, Hydrozoa) Invert Neurosci DOI 10.1007/s10158-010-0109-7)
The authors describe the molecular identity of apical and body tissue and the possible role of neurons in the organization of the nervous system between the apical and body domains, but they do not even mention the current work on the patterning of the Nematostella nervous system. They are encouraged to place their data in this larger development context as well.

Resubmission

First decision letter

MS ID#: DEVELOP/2022/200833

MS TITLE: Molecular and cellular architecture of the larval sensory organ in the cnidarian Nematostella vectensis

AUTHORS: Eleanor Gilbert, Callum Teeling, Tatiana Lebedeva, Siffreya Pedersen, Nathan Chrismas, Grigory Genikhovich, and Vengamanaidu Modepalli

I have now received all the referees reports on the above manuscript, and have reached a decision. The referees' comments are appended below, or you can access them online: please go to BenchPress and click on the 'Manuscripts with Decisions' queue in the Author Area.

The overall evaluation is positive and we would like to publish a revised manuscript in Development, provided that the referees' comments can be satisfactorily addressed. Please attend to all of the reviewers' comments in your revised manuscript and detail them in your point-by-point response. If you do not agree with any of their criticisms or suggestions explain clearly why this is so. If it would be helpful, you are welcome to contact us to discuss your revision in greater detail. Please send us a point-by-point response indicating your plans for addressing the referee’s comments, and we will look over this and provide further guidance.In particular, please note and fully address the important comments of the Reviewers pointing out that the inclusion of your new experiment means that more specific methodological details must be included in the MS, and that contextualizing the interpretations in the context of what is known, or what you hypothesize, about the role of the apical organ in Nematostella.

Reviewer 1

Advance summary and potential significance to field

Molecular and cellular architecture of the larval sensory organ in the cnidarian Nematostella vectensis

This is a resubmission of a manuscript on the apical organ in Nematostella. Tatiana Lebedeva and Grigory Genikovich have joined as new authors and first author is now Eleanor Gilbert. As a major addition to the manuscript, an experiment on the expression pattern and regulation of the homeobox gene ISX, isolated in their screen for apical organ-specific genes, has been included. Otherwise, the title, format, most of the figures and the main sections of the text have remained largely unchanged. – As stated before, our knowledge of the apical organ is still quite limited. This ciliary sensory organ has been found in many pelagic marine larvae. It probably functions as a chemo/mechanosensory receptor for larval attachment and the initiation of metamorphosis, but the composition and function of its sensory cells, the neurotransmitters and organogenesis are the subject of ongoing research by several groups. Apical organs have been found in anthozoans, lophotrochozoans and hemichordates, but it is as yet unclear whether they have a common origin or whether they arose through convergent evolution. Thus, the topic is interesting and the authors

© 2022. Published by The Company of Biologists under the terms of the Creative Commons Attribution License (https://creativecommons.org/licenses/by/4.0/).

were encouraged to place their nice but merely descriptive data in a more functional development context.

The new experiments on ISX-like (NVE14554) have significantly improved the quality of the manuscript.
ISX-like shows a spot-like expression centrally in the apical organ, which is negatively regulated by beta-catenin signaling. This "spot domain" is complementary to the "ring domain" first described by Sinigaglia et al. (2015). The authors also show that inhibition of ISX-like leads to a complete loss of the apical tuft.
Since the formation of the apical tuft depends on FGFa1, the authors further tested whether ISX-like is upstream or downstream of the FGF signaling pathway. They show that the expression of ISX-like was abolished by incubating the embryos with the FGF receptor inhibitor SU5402 and the MEK inhibitor U0126 suggesting that the expression of ISX-like is positively controlled by the FGF signaling pathway. Thus, ISX-like is an FGF pathway-dependent transcription factor that is not only responsible for the formation of the apical tuft domain, but also represses the fate of neuronal rings in apical cells.

Comments for the author

see above

Reviewer 2

Advance summary and potential significance to field

In the manuscript “Molecular and cellular architecture of the larval sensory organ in the cnidarian Nematostella vectensis” the authors generated a transcriptome to uncover the molecular signature of the apical organ of a cnidarian. Their analysis discovered that the apical domain comprises at least 6 different cell types (and show compartmentalization within the apical organ).
Knocking down ISX-like, a PRD class homeobox gene, resulted in the loss of the apical tuft cells and expansion of the neural ring identity into the spot region. This is very interesting manuscript describing in great detail the apical organ architecture of the cnidarian Nematostella. The manuscript includes beautiful and important data (in situs, immunofluorescence, striking phenotype upon the knockdown of a single transcription factor), careful experimental design and differential expression analyses which will for sure be of interest to a broad readership and will significantly enhance our understanding of the molecular and cellular toolkit of one of the first apical organs (sensory organs) in the animal kingdom.

Nevertheless, there are concerns which need to be addressed prior to publication. In addition, the manuscript would benefit from a stronger abstract (see my comments below) and a more detailed discussion (see my comments below).

Comments for the author

Main text:
Page 2, line 36: the authors first mention the apical organ, while then mentioning the apical domain. This should be consistent or needs clarification.
Page 2, line 44: A strong closing/summarizing sentence in the abstract is missing Page 3, line 72: “The morphology of the apical organ in cnidarian larvae is comparable to those of bilaterian larvae.” Please provide here relevant examples.
Page 3, line 75: The authors should explain how different their findings are in comparison to reference [2] and discuss their results considering the previous findings [ref 2]. Which genes have been found previously, which genes have now been newly identified?
Page 5-6, line 151-179: This very long paragraph is used to describe their findings regarding the differential expression of GPCRs. How important is this for the paper? I suggest shortening or make a better case why this is important (it’s also not mentioned in the abstract)
Page 11, line 325-340: The conclusion part of the paper in general is VERY short and not very strong. For example, the authors mention: “The evolutionary origin of apical organs and whether the apical organ of ciliated larva across different phyla share homology or evolved convergent

© 2022. Published by The Company of Biologists under the terms of the Creative Commons Attribution License (https://creativecommons.org/licenses/by/4.0/).

remains to be solved." Here it would be highly informative to discuss what speaks for homology of the apical organ and what not. The authors should also discuss what the known function of the apical organ is in cnidarians (or/and at least speculate if no information is available) and what their finding presented here adds to the big picture.
Figures:
Figure 1A: Little bit more care should have been taken while drawing the lines in the phylogenetic tree.
Figure 1B: Ctenophores have an apical organ. The authors should consider adding this here (and also in the main text) or mention, why ctenophore apical organ was omitted.
Figure 2J: Please indicate ring vs spot in the different panels as it would Figure 3B: Unclear what techniques (ISH, IF) have been used to visualize the neuropeptides. Please indicate in the figure/figure legend (please also double check methods section if this has been described in enough detail)

Reviewer 3

Advance summary and potential significance to field

In this paper Gilbert et al., map the cell types enriched at the apical organ in Nematostella vectensis. They reveal the expression profile of the planula apical domain by performing transcriptomics on the apical tissue that was dissected from the rest of the larval body. They integrated their data to previously available single cell RNA seq data and identified 6 cell types. They also identified ISX-like to be an FGF-dependent transcription factor responsible for forming the apical tuft. This paper complements previous work on the cell type characterization of the apical pole organ and provides evidence that the ISX-like transcription factor regulates apical tuft formation by repressing the fate of neural ring cells in the same region.

Comments for the author

Essential additional information needed Methodology 1. How did the authors dissect the apical pole of the motile planula embryos?
Were drugs used? Do these drugs potentially affect the transcriptional gene profile?
Results 1. There appears to be a complex relationship between FGFa1 and FGFa2 and their effect on apical tuft formation. According to (Sinigaglia et al 2015) Morpholino against FGFa2 expression leads to expanded apical tuft and Morpholino against FGFa1 leads to elimination of the apical tuft. In this paper two separate drug treatments that block FGFa1 expression also blocked ISX-like expression. Is FGFa2 expression also blocked under these drug treatments? Ideally, since these drug treatments may not be specific for either of the two FGF gene expression, it will be more precise to see the apical tuft and ISX-like expression in FGFa2 and FGFa1 morphants or shRNA knockdown mutants. Is ISX-like expression expanded in the former and eliminated in the latter? This work is important for concluding that ISX-like is an FGF signaling -dependent transcription factor and puts the new data of the paper in context with what is known in the field.
2. ISX-like expression appears to be significantly reduced in ISX-like sh RNA knockdown embryos. The authors deduce that the ring like gene expression is expanded in these mutants. Since the authors have successfully generated beautiful double in situs (Fig 6), a double in situ of ISX-like and selected ring genes in ISX-like sh RNA mutant embryos will be very useful and striking. Such an experiment eliminates the discrepancy of comparing in situs of different genes in different embryos collected at different times.
Minor comments 1. Since ISX-like shRNA procedure seems to be more efficient in reducing ISX-like levels compared to the Morpholino approach, what were the metamorphosis success numbers in ISX-like sh RNA knockdown embryos?
2. What is the embryonic stage the ISX-like knockdown in situs were performed in Fig 7?
3. Show the ISX-like expression in ISX-like shRNA knockdown embryos in main figures (move Fig S3A from Supp. Info into the main text).
4. Pattern of NVE14554 looks different between the two planula embryos in Fig 6J and Fig 6K, explain.
5. Methodology: qPCR primer sequences are missing DMSO concentration for drug treatments is missing

© 2022. Published by The Company of Biologists under the terms of the Creative Commons Attribution License (https://creativecommons.org/licenses/by/4.0/).

6. It will be useful to keep gene names consistent, for example replace NVE14554 with ISX-like throughout the paper text and figures. It looks like the last 2 figures of the paper were attached without proper integration into the manuscript.

First revision

Author response to reviewers' comments

We were happy to learn that all three reviewers found our work interesting and we appreciate the effort they made in order to improve the manuscript. We followed most of the suggestions made by the reviewers, added the relevant remarks and revised our manuscript accordingly. Please find below our point-by-point answers to the comments. We hope that you will find our revision satisfactory.

Best wishes,
Dr Vengamanaidu Modepalli
Dr Grigory Genikhovich

Reviewer 1 Advance Summary and Potential Significance to Field:
Molecular and cellular architecture of the larval sensory organ in the cnidarian Nematostella vectensis

This is a resubmission of a manuscript on the apical organ in Nematostella. Tatiana Lebedeva and Grigory Genikovich have joined as new authors and first author is now Eleanor Gilbert. As a major addition to the manuscript, an experiment on the expression pattern and regulation of the homeobox gene ISX, isolated in their screen for apical organ- specific genes, has been included. Otherwise, the title, format, most of the figures and the main sections of the text have remained largely unchanged. – As stated before, our knowledge of the apical organ is still quite limited. This ciliary sensory organ has been found in many pelagic marine larvae. It probably functions as a chemo/mechanosensory receptor for larval attachment and the initiation of metamorphosis, but the composition and function of its sensory cells, the neurotransmitters and organogenesis are the subject of ongoing research by several groups. Apical organs have been found in anthozoans,lophotrochozoans and hemichordates, but it is as yet unclear whether they have a common origin or whether they arose through convergent evolution. Thus, the topic is interesting and the authors were encouraged to place their nice but merely descriptive data in a more functional development context.

The new experiments on ISX-like (NVE14554) have significantly improved the quality of the manuscript. ISX-like shows a spot-like expression centrally in the apical organ, which is negatively regulated by beta-catenin signaling. This "spot domain" is complementary to the "ring domain" first described by Sinigaglia et al. (2015). The authors also show that inhibition of ISX-like leads to a complete loss of the apical tuft. Since the formation of the apical tuft depends on FGFa1, the authors further tested whether ISX-like is upstream or downstream of the FGF signaling pathway. They show that the expression of ISX-like was abolished by incubating the embryos with the FGF receptor inhibitor SU5402 and the MEK inhibitor U0126, suggesting that the expression of ISX-like is positively controlled by the FGF signaling pathway. Thus, ISX-like is an FGF pathway-dependent transcription factor that is not only responsible for the formation of the apical tuft domain, but also represses the fate of neuronal rings in apical cells.

Reviewer 1 Comments for the Author:
see above
Response: We thank Reviewer 1 for the positive evaluation of our work

Reviewer 2 Advance Summary and Potential Significance to Field:
In the manuscript “Molecular and cellular architecture of the larval sensory organ in the cnidarian Nematostella vectensis” the authors generated a transcriptome to uncover the molecular signature of the apical organ of a cnidarian. Their analysis discovered that the apical domain

© 2022. Published by The Company of Biologists under the terms of the Creative Commons Attribution License (https://creativecommons.org/licenses/by/4.0/).

comprises at least 6 different cell types (and show compartmentalisation within the apical organ). Knocking down ISX-like, a PRD class homeobox gene, resulted in the loss of the apical tuft cells and expansion of the neural ring identity into the spot region. This is very interesting manuscript describing in great detail the apical organ architecture of the cnidarian Nematostella. The manuscript includes beautiful and important data (in situs, immunofluorescence, striking phenotype upon the knockdown of a single transcription factor), careful experimental design and differential expression analyses which will for sure be of interest to a broad readership and will significantly enhance our understanding of the molecular and cellular toolkit of one of the first apical organs (sensory organs) in the animal kingdom.

Nevertheless, there are concerns which need to be addressed prior to publication. In addition, the manuscript would benefit from a stronger abstract (see my comments below) and a more detailed discussion (see my comments below).

Reviewer 2 Comments for the Author:
Main text:
Page 2, line 36: the authors first mention the apical organ, while then mentioning the apical domain. This should be consistent or needs clarification.
Response: line 85: We apologise if the terms are confusing. We term the whole Six3/6 and FoxQ2a-expressing area located aborally of the Wnt2 expression domain in the late gastrula “aboral” or “apical” domain – see Lebedeva et al., 2021. Apical organ forms in the centre of the apical domain in the mid-planula. It consists of ISX-like expressing cells surrounded by a ring of neurons. We now corrected the manuscript for consistency.

Page 2, line 44: A strong closing/summarising sentence in the abstract is missing
Response: line 40-43: We restructured the abstract and added a closing sentence.

Page 3, line 72: “The morphology of the apical organ in cnidarian larvae is comparable to those of bilaterian larvae.” Please provide here relevant examples.
Response: line 70: We included the relevant references

1. Sinigaglia, C., et al. (2013). "The Bilaterian Head Patterning Gene six3/6 Controls Aboral Domain Development in a Cnidarian."
2. Sinigaglia, C., et al. (2015). Molecular characterisation of the apical organ of the anthozoan Nematostella vectensis.
3. Marlow, H., et al. (2014). Larval body patterning and apical organs are conserved in animal evolution.
4. Nielsen, C. (2015). "Larval nervous systems: true larval and precocious adult.

Page 3, line 75: The authors should explain how different their findings are in comparison to reference [2] and discuss their results considering the previous findings [ref 2]. Which genes have been found previously, which genes have now been newly identified?
Response: line 122-124: We agree with this comment and provide the relevant analysis in the revised manuscript.
“We then overlapped our RNA-Seq-based dataset with the previously published list of 78 genes with confirmed aboral expression [2], and found that 71 out 78 were present among the 1185 aborally enriched transcripts we identified **(Supplementary Table 1)**.”

Page 5-6, line 151-179: This very long paragraph is used to describe their findings regarding the differential expression of GPCRs. How important is this for the paper? I suggest shortening or make a better case why this is important (it’s also not mentioned in the abstract)
Response: The GPCRs are certainly important candidates for understanding the signalling in the larval sensory system. Despite their importance, our understanding of non-bilaterian GPCRs is minimal. The GPCR data we present here is a valuable resource for the future exploration of their functional role in the larval sensory system. However, in the present state, since we only performed phylogenetic analysis, we did not want to emphasise these findings by putting them into the abstract.
In general, we agree with the reviewer's comments. To streamline the manuscript, we have shortened the results to key outcomes and moved the GPCR figure C & D to the Supplementary figure 2.

© 2022. Published by The Company of Biologists under the terms of the Creative Commons Attribution License (https://creativecommons.org/licenses/by/4.0/).

Page 11, line 325-340: The conclusion part of the paper in general is VERY short and not very strong. For example, the authors mention: “The evolutionary origin of apical organs and whether the apical organ of ciliated larva across different phyla share homology or evolved convergent remains to be solved.” Here it would be highly informative to discuss what speaks for homology of the apical organ and what not. The authors should also discuss what the known function of the apical organ is in cnidarians (or/and at least speculate if no information is available) and what their finding presented here adds to the big picture.

Response: line 317-356: Thanks for this extremely valid criticism. We completely restructured our conclusion in a new “Conclusions and outlook” section.

Figures:
Figure 1A: Little bit more care should have been taken while drawing the lines in the phylogenetic tree.
Response: We adjusted the lines.

Figure 1B: Ctenophores have an apical organ. The authors should consider adding this here (and also in the main text) or mention, why ctenophore apical organ was omitted. **Response:** Thanks for the comment! The aboral sensory organ of ctenophores is often termed “apical organ” due to its localisation at the aboral pole and its sensory function. However, the ctenophore apical organ is not a larval structure. Ctenophores are direct developers, whose “cydippid larva” is not a larva at all. It is a juvenile with a body plan very similar to that of an adult. The apical organ of the cydippid “larva” persists in the adult.
Moreover, in terms of anatomy, ctenophore apical organs are drastically different and clearly not homologous to the apical organs of Cnidaria and Bilateria. In contrast to the cnidarian and bilaterian statocysts, which are always cavities within the body of an animal, the ctenophore statocyst is a collection of lithocytes carrying large calcareous concretions suspended on four crescent-like springs made of 150-200 fused mechanosensory cilia and enclosed within a dome of modified cilia, i.e. it is “outside” of the body of the animal. Due to these reasons we omitted the Ctenophora apical organ in the original manuscript.
However, in order to prevent misinterpretation by readers, we now added a clarifying sentence in the legend to Fig. 1A: “Since ctenophore aboral sensory organs, which are sometimes also termed “apical organs”, are clearly not homologous to the larval apical organs of Cnidaria and Bilateria [77, 78] we do not include them on this Figure.”

Figure 2J: Please indicate ring vs spot in the different panels as it would
Response: The spot and ring genes are now separated into two panels and indicated as J (Ring genes) and K (Spot genes).

Figure 3B: Unclear what techniques (ISH, IF) have been used to visualise the neuropeptides. Please indicate in the figure/figure legend (please also double check methods section if this has been described in enough detail)
Response: We re-wrote the legend for the Fig. 6 to clarify this. Corresponding sections in the methods already existed.

Reviewer 3 Advance Summary and Potential Significance to Field:
In this paper Gilbert et al., map the cell types enriched at the apical organ in Nematostella vectensis. They reveal the expression profile of the planula apical domain by performing transcriptomics on the apical tissue that was dissected from the rest of the larval body. They integrated their data to previously available single cell RNA seq data and identified 6 cell types. They also identified ISX-like to be an FGF-dependent transcription factor responsible for forming the apical tuft. This paper complements previous work on the cell type characterisation of the apical pole organ and provides evidence that the ISX-like transcription factor regulates apical tuft formation by repressing the fate of neural ring cells in the same region.

Reviewer 3 Comments for the Author:
Essential additional information needed

© 2022. Published by The Company of Biologists under the terms of the Creative Commons Attribution License (https://creativecommons.org/licenses/by/4.0/).

Methodology
1. How did the authors dissect the apical pole of the motile planula embryos? Were drugs used? Do these drugs potentially affect the transcriptional gene profile?

Response: line 366-376: We did not use any drugs for the isolation of the apical pole. The procedure is now described in detail in the "Microdissection of Nematostella apical organs" section.

Results

1. There appears to be a complex relationship between FGFa1 and FGFa2 and their effect on apical tuft formation. According to (Sinigaglia et al 2015) Morpholino against FGFa2 expression leads to expanded apical tuft and Morpholino against FGFa1 leads to elimination of the apical tuft. In this paper two separate drug treatments that block FGFa1 expression also blocked ISX-like expression. Is FGFa2 expression also blocked under these drug treatments? Ideally, since these drug treatments may not be specific for either of the two FGF gene expression, it will be more precise to see the apical tuft and ISX-like expression in FGFa2 and FGFa1 morphants or shRNA knockdown mutants. Is ISX-like expression expanded in the former and eliminated in the latter? This work is important for concluding that ISX-like is an FGF signaling -dependent transcription factor and puts the new data of the paper in context with what is known in the field.

Response: The relationship between FGFa1 and FGFa2 is very clear and has been described in detail in the 2008 paper of Rentzsch et al., (DOI: 10.1242/dev.020784). Briefly, *Nematostella* apical domain expresses two FGF ligands, FGFa1 and FGFa2, and a single FGF receptor FGFRa. FGFa1 signals via the FGFRa and maintains the expression of *FGFa1*, *FGFa2*, and *FGFRa*. Morpholino knockdown of FGFa1 or FGFRa abolishes *FGFa1, FGFa2, and FGFRa* expression, as well as leads to the failure of the apical organ to form. In contrast, FGFa2 knockdown increases *FGFa1*, *FGFa2*, and *FGFRa* expression, and results in the formation of the much larger apical organ. Finally, a combined knockdown of FGFa2 and FGFRa or treatment of the FGFa2 morphants with the FGF receptor inhibitor SU5402 phenocopies the effect of the FGFa1 or FGFRa knockdown. Taken together, the data of Rentzsch et al. strongly suggest that: 1) FGFa1-mediated signalling via FGFRa is required for the formation of the apical organ; 2) FGFa1-mediated signalling via FGFRa is required for the expression of *FGFa1*, *FGFRa*, as well as the second aboral FGF ligand – *FGFa2*; 3) FGFa2 acts as a dominant-negative ligand preventing the uncontrolled expansion of the apical domain.
Both inhibitors used in this study (as well as in Rentzsch et al., 2008) act downstream of FGF ligands – either at the level of FGF receptor (SU5402) or at the level of activation of ERK (U0126), and thus they do not discriminate between different FGF ligands. Given that *FGFa1* expression is abolished upon U0126 treatment (Rentzsch et al., 2008), and that morpholino knockdowns of FGFa1 or FGFRa lead to a loss of *FGFa2* expression (Rentzsch et al., 2008), we consider it logical to expect that *FGFa2* expression will also be abolished once FGFRa is inhibited by SU5402 or ERK activation is inhibited by U0126.
However, to address the Reviewer's concern, we experimentally tested this by performing SU5402 and U0126 treatment and staining for *FGFa2*. The result was exactly as expected – *FGFa2* expression was abolished in both treatments (see the updated Supplementary Fig. 6). Given the clarity of the outcome of the pharmacological experiments (i.e. that *ISX-like* expression disappears upon the FGFR and MEK inhibitor treatments), additional knockdowns of the two FGF ligands are, in our opinion, unnecessary for the conclusion that *ISX-like* expression is FGF-dependent.

2. ISX-like expression appears to be significantly reduced in ISX-like sh RNA knockdown embryos. The authors deduce that the ring like gene expression is expanded in these mutants. Since the authors have successfully generated beautiful double in situs (Fig 6), a double in situ of ISX-like and selected ring genes in ISX-like sh RNA mutant embryos will be very useful and striking. Such an experiment eliminates the discrepancy of comparing in situs of different genes in different embryos collected at different times.

Response: Fig. 7C is showing the embryos from the same respective batch collected at the same time. The same is true for Fig S5C. The in situs clearly and consistently show the expression of the ring genes *NVE8226* and *NVE14902* in the apical patch domain – this is not our deduction but

© 2022. Published by The Company of Biologists under the terms of the Creative Commons Attribution License (https://creativecommons.org/licenses/by/4.0/).

an experimentally proven fact. We do not think there is any discrepancy here, and thus we do not see the need for performing double in situs.

Minor comments

1. Since ISX-like shRNA procedure seems to be more efficient in reducing ISX-like levels compared to the Morpholino approach, what were the metamorphosis success numbers in ISX-like sh RNA knockdown embryos?

Response: In gene knockdown approaches, the goal is to reduce the level of the protein of interest – e.g. by reducing the amount of the mRNA encoding it (RNAi) or by interfering with the translation of this mRNA (translation-blocking morpholino). Thus, we do not expect any effect of the ISX-like translation morpholino on the *ISX-like* mRNA quantity, as long as there is no autoregulatory effect of ISX-like protein on the transcription of the *ISX-like* gene. On the other hand, RNAi of ISX-like directly reduces the amount of *ISX-like* transcript in the embryo. In the absence of the specific anti-ISX-like antibody, which would allow us to measure ISX- like protein amount, we cannot quantitatively compare the efficiencies of the morpholino- mediated and shRNA-mediated ISX-like knockdown. However, in our extensive experience, RNAi is usually a less efficient knockdown method than a translation morpholino-based knockdown. Figure 7A shows that already by 3 dpf, the *ISX-like* mRNA quantity is back to approximately 60-70% of the normal level based on qPCR and in situ, while metamorphosis starts around 6-7 dpf. Therefore, we did not expect to see an effect of the ISX-like RNAi on the success of the metamorphosis, and hence chose a potentially longer-lasting morpholino- mediated knockdown to assess the role of ISX-like in metamorphosis.
However, we followed the Reviewer's suggestion and tested whether RNAi of ISX-like affected metamorphosis on three biological replicates. As expected, the differences in the metamorphosis rates were insignificant (74% in shControl vs. 72% in shISX-like, p=0.413383). We do not include these data in the revised version of the manuscript.

2. What is the embryonic stage the ISX-like knockdown in situs were performed in Fig 7?

Response: We included the age of the embryos into the legends of Fig. 7 and Fig. 8.

3. Show the ISX-like expression in ISX-like shRNA knockdown embryos in main figures (move Fig S3A from Supp. Info into the main text).

Response: We thank the Reviewer for this comment. We moved the analysis of the ISX-like expression in shISX-like from the supplement and integrated it into the figure 7. The analysis of the early normal expression of ISX-like was moved to the supplement instead.

4. Pattern of NVE14554 looks different between the two planula embryos in Fig 6J and Fig 6K, explain.
Response: The pattern of *NVE14554* is the same in both Fig 6J and K; they look slightly different, as the presented 2D images are in different Z axis/depths. To avoid such misperception, we provided detailed and higher magnification images on Fig 6L and Fig 6M and in the Supplementary Movies 5 and 6.

5. Methodology: qPCR primer sequences are missing. DMSO concentration for drug treatments is missing.
Response: Thank you. We included the primer sequences and DMSO concentration in the Methods section.

6. It will be useful to keep gene names consistent, for example replace NVE14554 with ISX- like throughout the paper text and figures. It looks like the last 2 figures of the paper were attached without proper integration into the manuscript.
Response: We updated the gene names as NVE14554 (ISX-like) where necessary.

© 2022. Published by The Company of Biologists under the terms of the Creative Commons Attribution License (https://creativecommons.org/licenses/by/4.0/).

Second decision letter

MS ID#: DEVELOP/2022/200833

MS TITLE: Molecular and cellular architecture of the larval sensory organ in the cnidarian Nematostella vectensis

AUTHORS: Eleanor Gilbert, Callum Teeling, Tatiana Lebedeva, Siffreya Pedersen, Nathan Chrismas, Grigory Genikhovich, and Vengamanaidu Modepalli
ARTICLE TYPE: Research Article

I am happy to tell you that your manuscript has been accepted for publication in Development, pending our standard ethics checks.

© 2022. Published by The Company of Biologists under the terms of the Creative Commons Attribution License (https://creativecommons.org/licenses/by/4.0/).
